# Supplementary material for: Interpretable online network dictionary learning for inferring long-range chromatin interactions
Source: PLoS Comput Biol. 2024 May 16;20(5):e1012095. doi: 10.1371/journal.pcbi.1012095 (PMC11135774; doi:10.1371/journal.pcbi.1012095)
Supplement: S1 Text — Supplemental material, including figures and tables, is available in the Supplement file. The online cvxNDL code and test datasets are available at: https://urldefense.com/v3/__https://github.com/rana95vishal/chromatin_DL/__;!!DZ3fjg!4VWHhuROFHcJ1bWTZ8pNxUn75T-K3BfsdTvxM1iU1hXmSGX84JcRsXyIZZS0k5Iaub9yNiansT9FS12EO52_XsbpA_s$. A tool that enables readers with color-blindness to view the images using a more appropriate color palette is described at the end of the Supplement. (PDF) [file pcbi.1012095.s001.pdf]

Supplement - Interpretable Online Network Dictionary Learning for Inferring Long-Range Chromatin Interactions

Vishal Rana, Jianhao Peng, Chao Pan, Hanbaek Lyu, Albert Cheng, Minji Kim, Olgica Milenkovic

A Motivation

Dictionary learning (DL), a form of nonnegative matrix factorization (MF), has been widely used in the analysis of biological data. However, *efficient*, and *biologically interpretable* computational methods for analyzing long-distance multiplexed chromatin interactions at a single-cell level are still lacking. This gap exists primarily because classical DL methods are not tailored for network data analysis. Furthermore, these interactions cannot be easily visualized or predicted via classical clustering approaches. This issue is best illustrated by Fig A, where a part of the contact map contains three hidden clusters, colored red, green, and blue [1]. When using a linear chromatin order, the particular structure of the clusters is not observable. By rearranging the rows/columns, the cluster structure becomes apparent within the adjacency matrix.

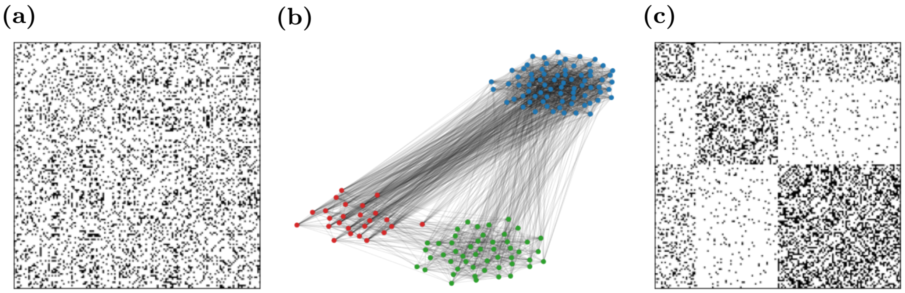

Fig A. (a) Observed adjacency matrix of a three-cluster model, where points are arranged in linear order with dense interactions existing both at short- and long-range. **(b)** The underlying cluster structure. **(c)** The reordered adjacency matrix that reveals all interaction classes.

To mitigate this issue, we propose a novel online convex network dictionary learning algorithm (online cvxNDL) that imposes "convexity" constraints on the sampled subgraph patterns to address the issue of interpretability. Furthermore, due to its online nature, it scales to large graph-structured datasets. The detailed algorithmic implementations are described in the next section.

B Algorithmic Details

The algorithms presented in this section describe the detailed steps of implementation outlined in the Methods Section.

B.1 MCMC Sampling of Subnetworks

We use the MCMC sampling in conjunction with subnetwork sampling to generate online samples. We seek samples in the form of subnetworks induced by k nodes in the original input network $\mathcal{G}$ such that these subnetworks contain the template F topology. Given an input network $\mathcal{G} = (V, \mathbf{A})$ and a template network $F = ([k], \mathbf{A}_F)$, we define a set of homomorphisms as a vector of the form (with the assumption that $0^0 = 1$):

$$\mathtt{Hom}(F, \mathcal{G}) = \left\{ \underline{x} : [k] \to [n] \,\middle|\, \prod_{1 \le i,j \le k} \mathbf{A}[\underline{x}[i], \underline{x}[j]]^{\mathbf{A}_F[i,j]} = 1 \right\}.$$

Algorithm A outlines how to use rejection sampling to obtain one homomorphism $\underline{x}$ (an illustrative example is presented in Fig 1(d) in the main text). In this work, we use a k-path as the template network, where a k-path represents a directed path from node 1 to k. Paths serve as a simple and natural choice for networks containing inherent long paths, such as chromatin interaction networks, where most contact measurements are due to proximity in the linear chromosome order.

Algorithm A Rejection Sampling of Homomorphisms

```
input: Network G = ([n], A), template F = ([k], A_F) (under the assumption that there exists at least one homomorphism F → G).
while true do
    Sample x = (x[1], x[2], ..., x[k]) ∈ [n]^k so that x[i]'s are i.i.d.
    if ∏_{1≤i,j≤k} A[x[i], x[j]]^{A_F[i,j]} > 0 then
        break
    end if
end while
return A homomorphism x : F → G.
```

While we can find different homomorphisms from the input $\mathcal{G}$ by iteratively executing Algorithm A, this method is computationally expensive. To efficiently generate a sequence of sample adjacency matrices $\mathbf{A}_{\underline{x}_t}$ from $\mathcal{G}$, the MCMC sampling algorithm gradually changes the sampled subnetwork based on previous samples as described in Algorithm B. An illustrative example is shown in Fig 1(e) in the main text. This sampling algorithm was introduced in [3, 4].

Algorithm B The MCMC Sampling Algorithm

input: Network $\mathcal{G} = ([n], \mathbf{A})$, template $F = ([k], \mathbf{A}_F)$, and one homomorphism $\underline{x} : F \to \mathcal{G}$.
Sample $v \in \text{Neighbor}(\underline{x}[1])$ with probability $P(v) = \frac{1}{\mathcal{N}[\underline{x}[1]]}$.
Compute the acceptance probability
$\beta = \min\left\{ \frac{\sum_{c\in[n]} A^{k-1}[v,c]}{\sum_{c\in[n]} A^{k-1}[\underline{x}[1],c]} \frac{\sum_{c\in[n]} A[\underline{x}[1],c]}{\sum_{c\in[n]} A[v,c]}, 1 \right\}$.
Sample u uniformly at random from $[0,1]$.
if $u < \beta$ **then**
  $\underline{x}'[1] = v$
else
  $\underline{x}'[1] = \underline{x}[1]$
end if
for $s = 2, 3, \ldots, k$ **do**
  Sample $w \in [n]$ with probability $P_s(w) = \frac{\mathbf{A}[\underline{x}'[s-1],w]}{\sum_{c\in V} \mathbf{A}[\underline{x}'[s-1],c]}$.
  $\underline{x}'[s] = w$
end for
return New homomorphism $\underline{x}' : F \to \mathcal{G}$.

B.2 Online Convex NDL (online cvxNDL)

Our online cvxNDL algorithm consists of two parts: initialization and iterative optimization. For initialization, we compute an initial choice for the dictionary elements $\mathbf{D}_0$ and initialize the representative regions $\hat{\mathbf{X}}_0^{(j)}$, $\forall j \in [K]$ using i.i.d. sampling of homomorphisms (Algorithm C). Note that we use i.i.d. sampling of homomorphisms only during the initialization step, and MCMC sampling afterwards. Upon initialization, we iteratively optimize the dictionary and the representative regions in the next phase (Algorithm D). The output of the latter algorithm is the final dictionary $\mathbf{D}_T$ and the corresponding representative regions for all dictionary elements $\hat{\mathbf{X}}_T^{(j)}$, $\forall j \in [K]$. Due to the added convexity constraint, each dictionary element $\mathbf{D}_T[:,j]$ at the final step T has the following interpretable form:

$$\mathbf{D}_T[:,j] = \sum_{i\in[N_j]} w_{j,i} \hat{\mathbf{X}}_T^{(j)}[:,i], \text{ s.t. } \sum_{i\in[N_j]} w_{j,i} = 1, w_{j,i} \geq 0, i \in [N_j], j \in [K].$$

The weight $w_{j,i}, i \in [N_j]$ is the convex coefficient of the ith representative of dictionary element $\mathbf{D}_T[:,j]$.

Algorithm C Initialization

1: **input:** Use rejection sampling in Algorithm A to sample i.i.d homomorphisms $\underline{x}_1, \underline{x}_2, \ldots, \underline{x}_N$.

2: For each homomorphism, define an adjacency matrix such that: $\mathbf{A}_{\underline{x}_i}[a, b] = \mathbf{A}[\underline{x}_i[a], \underline{x}_i[b]]$. Flatten the adjacency matrices into vectors: $\underline{x}_1, \underline{x}_2, \ldots, \underline{x}_N$, $\underline{x}_i \in \mathbb{R}^d, d = k^2$ and collect them in $\hat{\mathbf{X}} \in \mathbb{R}^{d \times N}$.

3: Run K-means on $\hat{\mathbf{X}}$ to generate the cluster indicator matrix $\mathbf{H} \in \{0, 1\}^{N \times K}$ and determine the initial cluster sizes (subsequent representative set sizes) $N_i, i \in [K]$.

4: Compute $\mathbf{D}_0$ and $\hat{\mathbf{X}}_0^{(i)} \in \mathbb{R}^{d \times N_i}$, $\forall i \in [K]$, according to:

$$\mathbf{D}_0 = \hat{\mathbf{X}} \, \mathbf{H} \, \mathrm{diag}(1/N_1, \ldots, 1/N_K)$$

and summarize the initial representative sets of the clusters into matrices $\hat{\mathbf{X}}_0^{(i)}$, $i = [K]$.

5: **return** $\mathbf{D}_0$, $\{\hat{\mathbf{X}}_0^{(i)}\}_{i \in [K]}$.

Algorithm D Online cvxNDL

1: **input:** Network $\mathcal{G} = ([n], \mathbf{A})$, template $F = ([k], \mathbf{A}_F)$, a parameter $\lambda \in \mathbb{R}$, max number of iterations T, and number of dictionary elements K.

2: **initialization:** Compute $\mathbf{D}_0$, $\{\hat{\mathbf{X}}_0^{(i)}\}_{i \in [K]}$ using Algorithm C. Set $\mathbf{A}_0 = \mathbf{0}$, $\mathbf{B}_0 = \mathbf{0}$.

3: **for** $t = 1$ to T **do**

4: MCMC sample a homomorphism $\underline{x}_t$ (Algorithm B). Find its adjacency matrix $\mathbf{A}_{\underline{x}_t}[a, b] = \mathbf{A}[\underline{x}_t[a], \underline{x}_t[b]]$ and flatten it to $\underline{x}_t$.

5: Update $\mathbf{\Lambda}_t$ according to:

$$\mathbf{\Lambda_t} = \underset{\mathbf{\Lambda} \in \mathbb{R}^{K \times 1}}{\arg\min} \frac{1}{2} \left\| \underline{x}_t - \mathbf{D}_{t-1} \mathbf{\Lambda} \right\|_2^2 + \lambda \left\| \mathbf{\Lambda} \right\|_1 . \tag{1}$$

6: Set $\mathbf{A}_t = \frac{1}{t}((t-1)\mathbf{A}_{t-1} + \mathbf{\Lambda}_t \mathbf{\Lambda}_t^T)$ and $\mathbf{B}_t = \frac{1}{t}((t-1)\mathbf{B}_{t-1} + \underline{x}_t \ \mathbf{\Lambda}_t^T)$.

7: Choose the index of the basis i_t to be updated according to $i_t = \arg\max_{j \in [k]} \mathbf{\Lambda}_t[j]$

8: Generate the augmented representative regions $\{\hat{\mathbf{Y}}_t^l\}_{l \in [N_{i_t}] \cup \{0\}}$:

$$\begin{aligned} \hat{\mathbf{Y}}_t^0 &= \hat{\mathbf{X}}_{t-1}^{i_t} \\ \{\hat{\mathbf{Y}}_t^l\}_{l \in [N_{i_t}]} : \ \hat{\mathbf{Y}}_t^l[j] &= \begin{cases} \hat{\mathbf{X}}_{t-1}^{i_t}[j], & \text{if } j \in [N_i] \setminus l \\ \underline{x}_t, & \text{if } j = l. \end{cases} \end{aligned} \tag{2}$$

9: Update $\{\hat{\mathbf{X}}_i^t\}_{i \in [K]}$ and $\mathbf{D}_t$ by executing the following two steps

- Compute $l^\star, \hat{\mathbf{D}}^\star$ by solving the optimization problems:

$$l^\star, \hat{\mathbf{D}}^\star = \underset{\substack{l, \ \mathbf{D} \ \text{ s.t.} \\ \mathbf{D}[j] \in \text{cvx}\{\hat{\mathbf{X}}_{t-1}^j\} \ j \neq i_t, \\ \mathbf{D}[i_t] \in \text{cvx}\{\hat{\mathbf{Y}}_t^l\}}}{\arg\min} \frac{1}{2} \operatorname{Tr}(\mathbf{D}^T \mathbf{D} \mathbf{A}_t) - \operatorname{Tr}(\mathbf{D}^T \mathbf{B}_t).$$

- Set

$$\begin{aligned} \hat{\mathbf{X}}_t^i &= \begin{cases} \hat{\mathbf{Y}}_t^{l^\star}, & \text{if } i = i_t \\ \hat{\mathbf{X}}_{t-1}^i, & \text{if } i \in [K] \setminus i_t, \end{cases} \\ \mathbf{D}_t &= \hat{\mathbf{D}}^\star. \end{aligned}$$

10: **end for**

11: **return** $\mathbf{D}_T$, $\hat{\mathbf{X}}_T^{(i)}$, $\forall i \in [K]$.

C Synthetic Data Analysis

We tested our online cvxNDL method on a network (graph) generated by Stochastic Block Model (SBM) [1], containing 150 nodes with 3 clusters of size $25, 50, 75$. Due to the small size of the synthetic set, we fixed the number of dictionary elements to $K = 6$ and used a path of length 11 as our template. In the initialization step, we sampled 30 subgraphs from the input synthetic data network, with each dictionary element represented by at least 3 representatives. The maximum number of iterations of the online method was set to $1,000$.

We compared online cvxNDL with various baseline methods, including NMF, CMF, and online NDL. The learned dictionary elements for different methods are shown in Fig B. The dictionary elements in online NDL and online cvxNDL are ordered by their importance score defined as $\gamma(i) = \frac{\mathbf{A}_t[i,i]^2}{\sum_{j\in[K]} \mathbf{A}_t[j,j]^2}$. Each square block in the subplots indicates one dictionary element in the form of an adjacency matrix. The color-shade reflects the values in the adjacency matrix, with black corresponding to 1 (the largest value) and white corresponding to 0 (the smallest value).

From the results, we can see that dictionaries generated using NMF only contain partial interaction structures and are hard to interpret. The two convex methods, CMF and online cvxNDL, contain the template structure in all learned dictionary elements and show stronger off-diagonal connectivity, which is expected as the input data has slightly stronger connections between the first and last cluster than other pairs (See Fig A). Online NDL dictionary elements represent "a middle ground" between NMF and online cvxNDL. Dictionary elements 2, 0, and 4 resemble those generated by NMF, while dictionary elements 1, 5, and 3 are similar to the ones generated by online cvxNDL, although with weaker connectivity. Also, the importance score distributions of online NDL and online cvxNDL differ substantially. In online NDL, dictionary element 1 in Fig Bb is the dominant component in representations, whereas, in online cxvNDL, the top two dictionary elements (dictionary elements 2 and 5 in Bd) share similar scores and the dictionary elements, in general, have a more balanced distribution of importance scores. From the original adjacency, we can see that there are indeed two different connectivity patterns in the network captured by online cvxNDL.

To show that our method scales well, and significantly better than regular convex methods, we generated synthetic data and compared the running time and computed memory requirements for online cvxNDL and convex matrix factorization (CMF), the only other method that provides biologically interpretable results. We created synthetic datasets of successively larger sizes using the stochastic block model with n = 500, 1000, 1500, 2000, and 2500 nodes, respectively. The graphs consist of 3 underlying clusters of sizes $0.2 \times n$, $0.2 \times n$, and $0.6 \times n$. Although these numbers appear rather small, CMF already becomes computationally prohibitive for several thousand nodes. It hence represents the bottleneck for comparative studies (see Fig C(a)). In terms of compute memory requirements, online cvxNDL seems to follow a near-constant trend (see

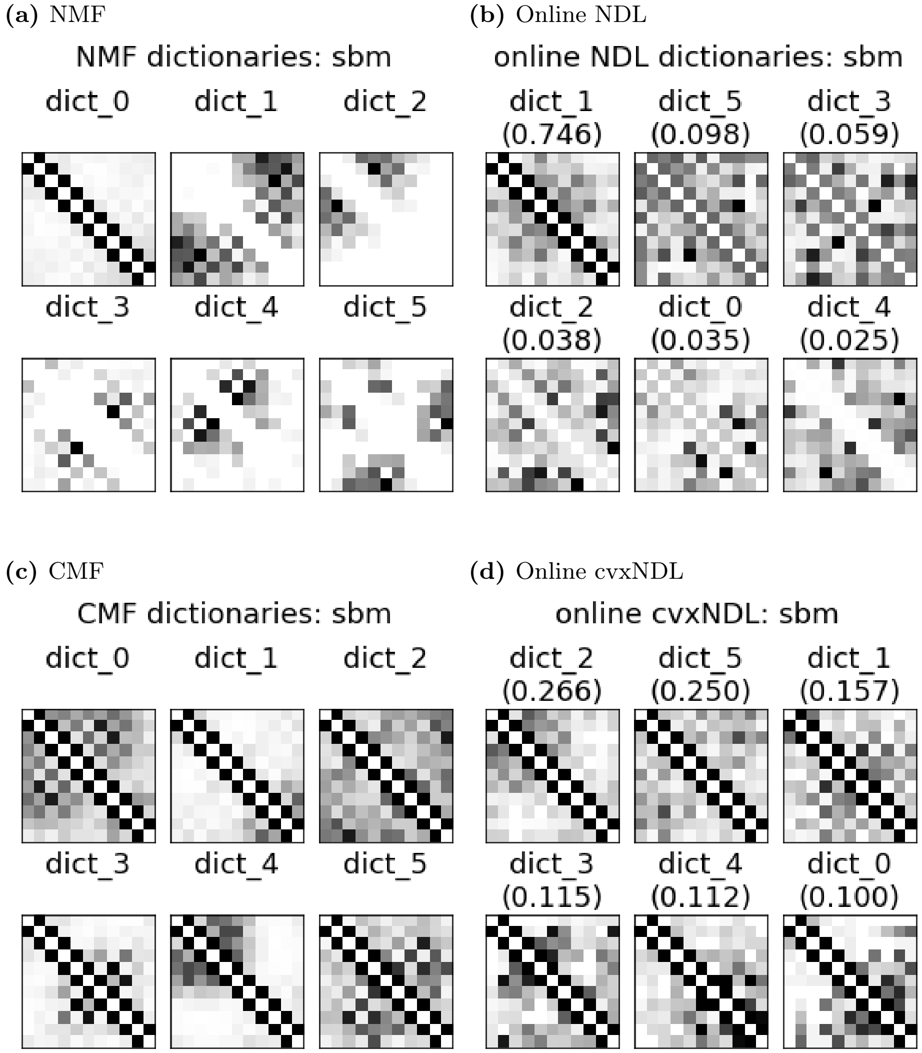

(a) NMF **(b)** Online NDL **(c)** CMF **(d)** Online cvxNDL

Fig B. Dictionary elements generated by different methods on an SBM synthetic dataset. Numbers in parenthesis are the importance scores for online NDL and online cvxNDL.

Fig C(b)). This is due to the fact that at each step, the online cvxNDL algorithm looks at one sample and decides to retain it or reject it based on the improvement it provides for the learned dictionaries. Hence, the compute memory scales with the size of the dictionary and not the size of the input network. CMF, on the other hand, requires access to the entire input graph for each update step.

(a) Run time **(b)** Compute memory

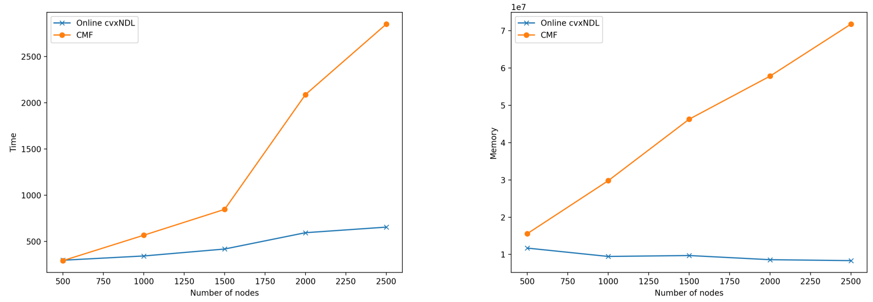

Fig C. Run time and memory requirements for CMF and online cvxNDL for successively larger networks generated using SBM.

Reconstruction accuracy: To validate the reliability of our learned dictionaries for representing the global interactions, we reconstructed the whole graph by aggregating the regenerated subgraphs: $\hat{\mathbf{x}}_i = \mathbf{D}_T \underline{\alpha}_i$ from the same MCMC sampling stream. For each method we selected the top-m edges after aggregation to reconstruct the original adjacency matrix, where m is the number of edges in the original adjacency matrix. The original and the reconstructed adjacency matrices are shown in Fig 7 in the main text. For comparison, we also added the reconstructed adjacency achieved when using random dictionary elements. From the results, we can see that all baseline methods, as well as online cvxNDL, almost perfectly reconstruct the original network, while, clearly random dictionaries do not capture any meaningful information. We also report the average precision recall score for each method, both for synthetic and real datasets as listed in Table 1 in the main text.

D ChIA-Drop Dataset

The preprocessed and binned RNAPII ChIA-Drop data includes $45,938$, $42,292$, $49,072$, and $55,795$ nodes and $36,140$, $28,387$, $53,006$, $45,530$ hyperedges for chromosome chr2L, chr2R, chr3L and chr3R respectively. The size distribution of hyperedges is given in Table A. The clique-expanded input network has $113,606$, $85,316$, $161,590$, and $143,370$ edges respectively. Fig D plots the number of MCMC samples needed for given percentages of node coverage.

The dictionary elements for each of the 4 chromosomes are presented in Fig 5 in the main text. The density or complexity of dictionary elements, defined as $\rho = \frac{1}{k^2}\sum_{i,j=1}^{k} \mathbf{D}_T[i,j]$, is reported in Table B while the median distance of pairwise interacting nodes in all representatives of a dictionary element is reported in Table C.

Table A. Number of hyperedges of various sizes observed in the ChIA-Drop data for various chromosomes.

hyperedge sizes	chr2L	chr2R	chr3L	chr3R
2	28373	22951	42175	35585
3	5723	4018	8103	7379
4	1307	936	1804	1700
5	424	275	533	479
6	136	94	196	187
7	60	41	82	69
8	48	29	38	31
9	21	15	28	22
10	8	5	16	7
11	7	6	9	8
12	11	2	7	9
13	5	2	5	7
14	7	2	2	5
15	4	2	1	4
16	3	2	1	4
17	1	2	2	0
18	2	1	1	1
19	0	1	0	0
$\geq$20	1	4	4	7

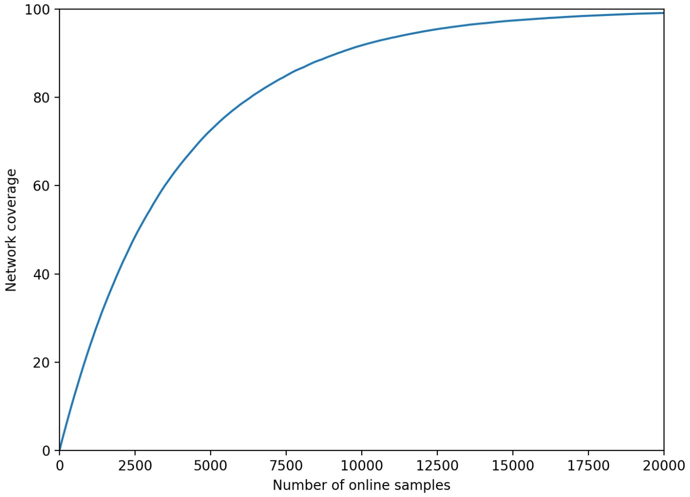

Fig D. Number of MCMC samples needed for given percentages of node coverage.

Table B. Density of dictionary elements, reported for all chromosomes.

Dictionary element	chr2L	chr2R	chr3L	chr3R
1	0.146	0.158	0.168	0.161
2	0.188	0.165	0.156	0.157
3	0.134	0.185	0.141	0.140
4	0.220	0.147	0.159	0.179
5	0.145	0.146	0.142	0.139
6	0.132	0.297	0.148	0.173
7	0.162	0.189	0.191	0.184
8	0.158	0.184	0.164	0.147
9	0.148	0.136	0.210	0.183
10	0.177	0.166	0.168	0.157
11	0.220	0.261	0.163	0.161
12	0.168	0.162	0.145	0.157
13	0.204	0.203	0.186	0.142
14	0.225	0.142	0.148	0.205
15	0.142	0.229	0.262	0.163
16	0.173	0.184	0.143	0.205
17	0.189	0.263	0.127	0.224
18	0.161	0.219	0.152	0.251
19	0.182	0.159	0.183	0.242
20	0.187	0.156	0.170	0.193
21	0.231	0.157	0.199	0.126
22	0.143	0.195	0.165	0.150
23	0.162	0.201	0.134	0.175
24	0.223	0.141	0.167	0.212
25	0.167	0.212	0.140	0.208

Table C. Median distance of pairwise interacting nodes within each dictionary element and for each chromosome.

dictionary element	chr2L	chr2R	chr3L	chr3R
1	10758	6738	7328	14753
2	8523	7688	12934	14760
3	9906	8759	9539	12666
4	8354	7158	12690	11748
5	9847	7651	10412	13674
6	8547	6953	10608	15598
7	10024	9383	11994	13498
8	8870	9226	10399	12830
9	10692	7085	14414	12493
10	11220	6414	9466	11930
11	10455	10711	10130	11421
12	8488	7656	11694	9398
13	9979	7706	14206	13455
14	10591	8251	8689	12540
15	10928	7284	10532	12572
16	10268	7143	8849	13842
17	8545	9681	9978	15184
18	8675	6859	8558	11974
19	9854	7882	8501	18233
20	9314	8199	10532	11592
21	9343	8872	9728	12791
22	8105	6418	10214	13301
23	8870	7418	11012	14239
24	9527	8764	10010	12692
25	11072	9711	13471	11316

D.1 Results for Baseline Methods Applied to ChIA-Drop Datasets

(a) chr2L

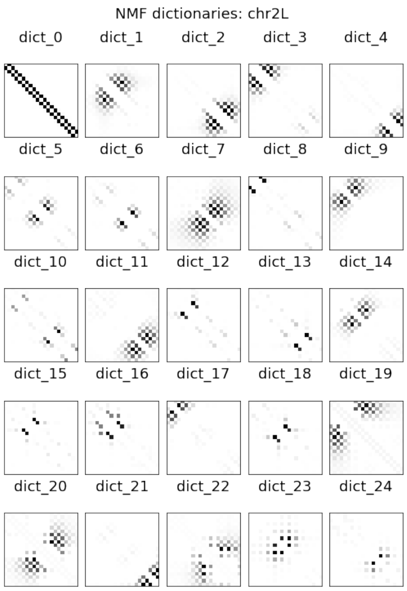

(b) chr2R

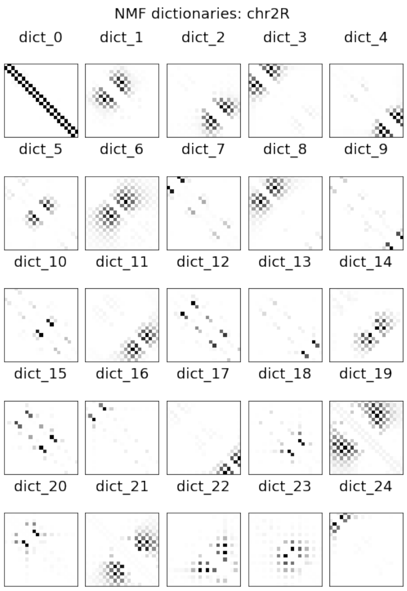

(c) chr3L

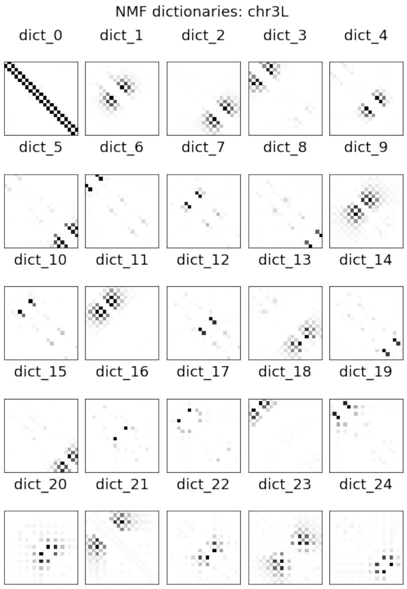

(d) chr3R

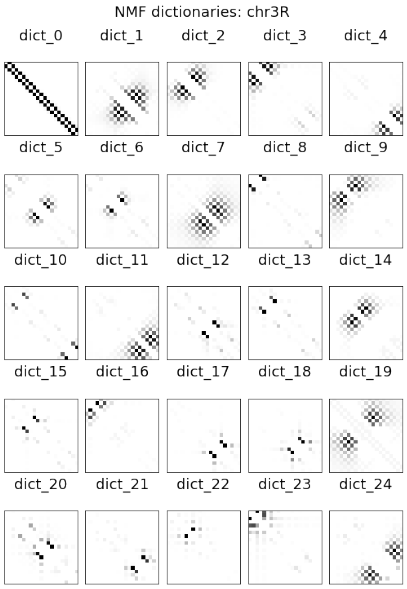

Fig E. Dictionaries learned by NMF for chr2L, 2R, 3L and 3R.

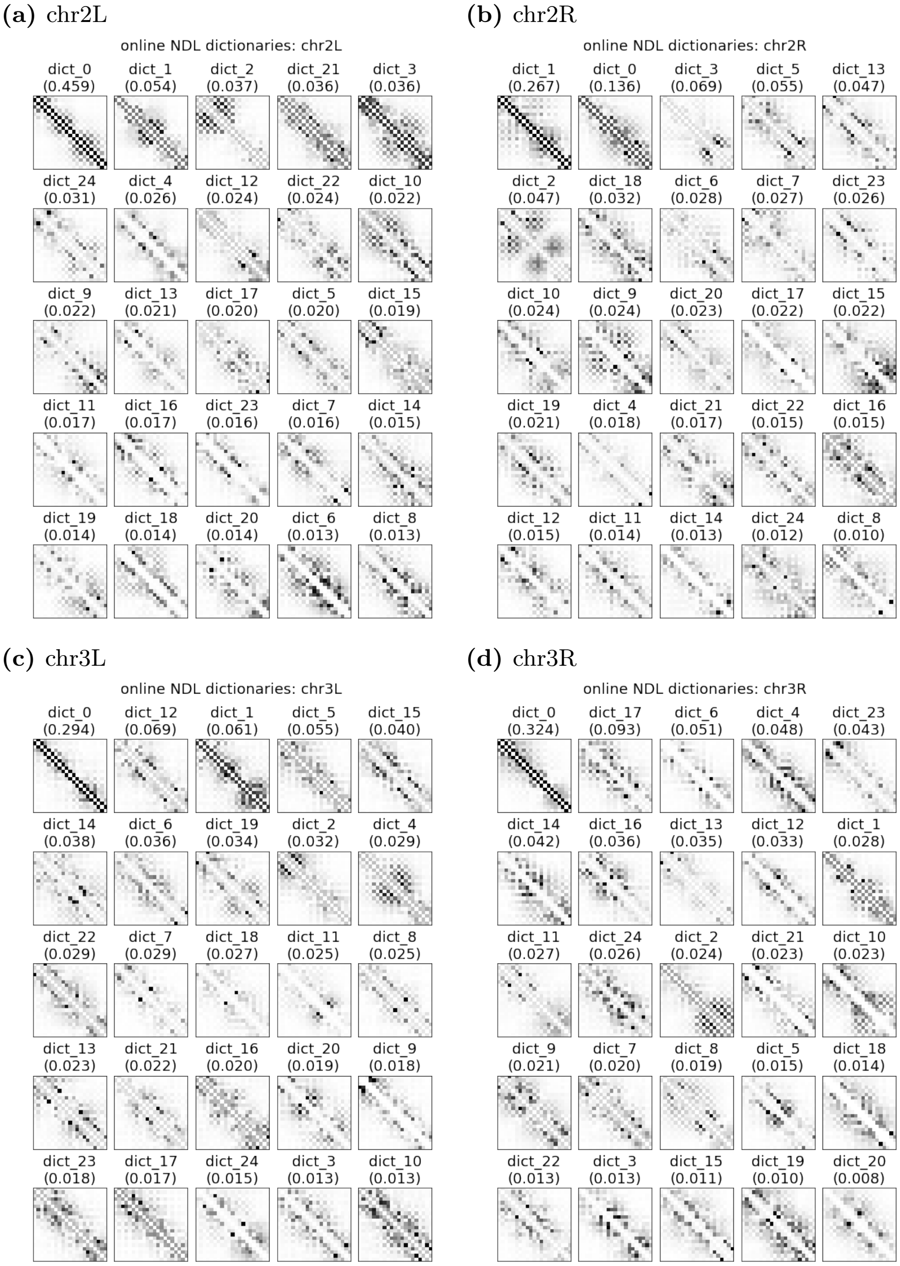

Fig F. Dictionaries learned by online NDL for chr2L, 2R, 3L and 3R.

(a) chr2L

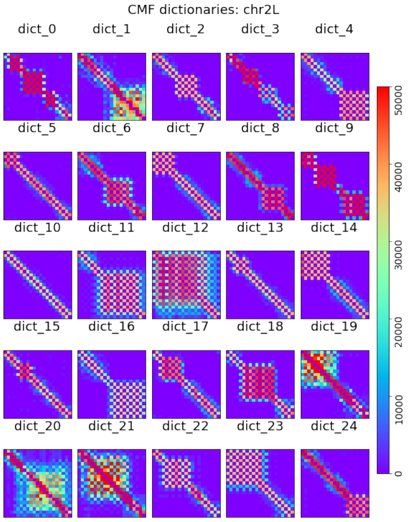

(b) chr2R

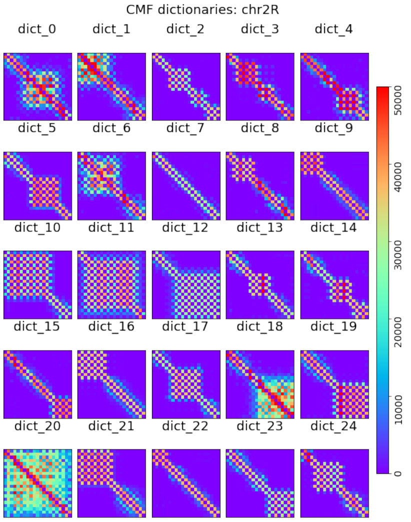

(c) chr3L

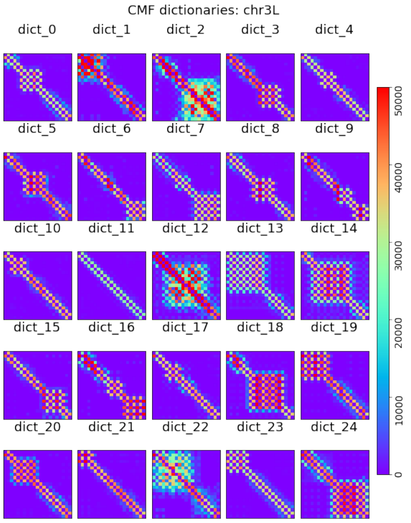

(d) chr3R

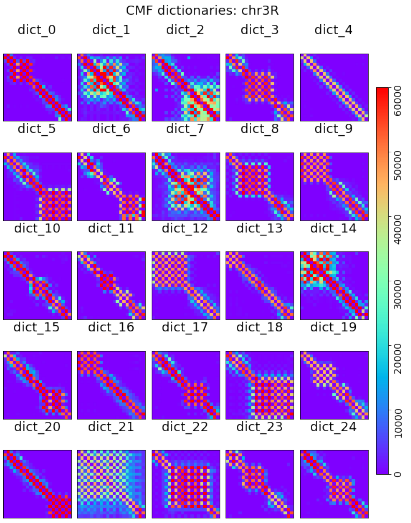

Fig G. Dictionaries learned by CMF for chr2L, 2R, 3L and 3R.

E Reconstruction of ChIA-Drop Contact Maps

The reconstructions for 4 randomly selected subnetwork samples are shown in Fig H, providing a means to visually assess the accuracy of reconstructed small-scale interactions. While all dictionary learning methods have comparable reconstruction accuracy, random dictionary elements fail to reconstruct the original subnetwork.

(a) Reconstruction of sample #15657

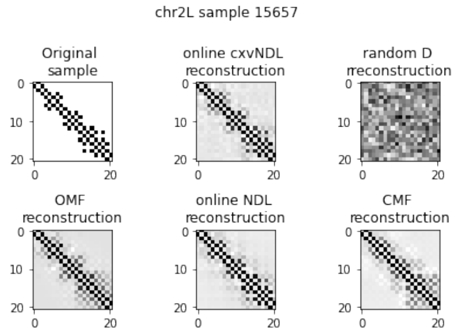

(b) Reconstruction of sample #8814

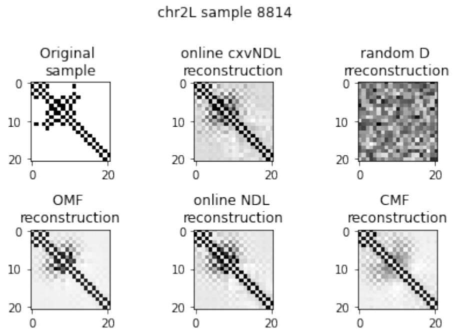

(c) Reconstruction of sample #2019

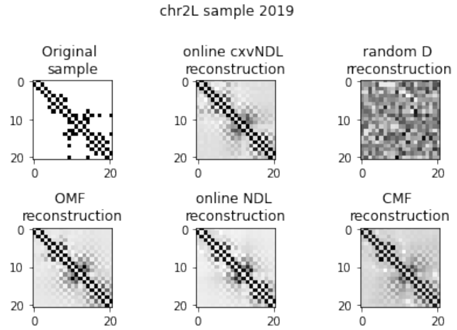

(d) Reconstruction of sample #9632

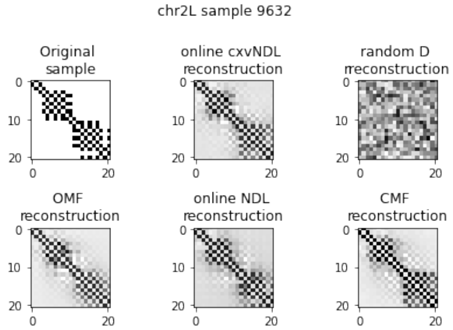

Fig H. Reconstructed adjacency matrices for subnetwork samples from chr2L obtained using different methods and random dictionary elements. OMF stands for Ordinary (Standard) MF or NMF.

We also compared the reconstruction accuracy of various methods, including random dictionary models, for the complete networks corresponding to the four chromosomes, chr2L, chr2R, chr3L, and chr 3R. The corresponding reconstruction accuracy values are reported in Table 1 of the main text. The table clearly shows that random dictionaries offer low reconstruction accuracy. The corresponding reconstructed networks, along with the original network, are shown in Figs I- L.

The random dictionary elements used in Figs I- L are obtained by randomly selecting a subset of MCMC sampled k-paths. However, k-paths will capture some local interaction patterns even when selected randomly. This is reflected

by the "acceptable" partial reconstruction of the contact maps via random dictionaries. Even for chromosome 3L (Fig K), for which the reconstruction results look visually indistinguishable from the original, the reconstruction accuracy is only 52%. In comparison, our method provides significantly higher reconstruction accuracy for all 4 chromosomes. Additionally, our method also offers excellent accuracy on synthetic datasets of variable node sizes.

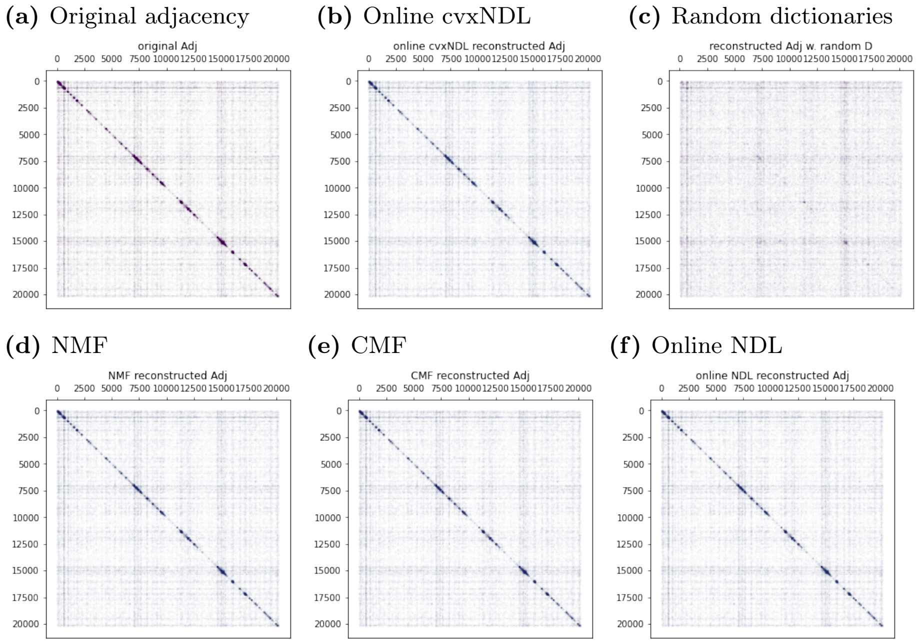

Fig I. Comparison of network reconstructions obtained using different baseline methods and random dictionaries for *Drosophila* chromosome 2L. (a): The original adjacency matrix; (b, c, d, e, f): Reconstructed network adjacency matrices with online cxvNDL, random dictionary elements, NMF, CMF and online NDL, respectively.

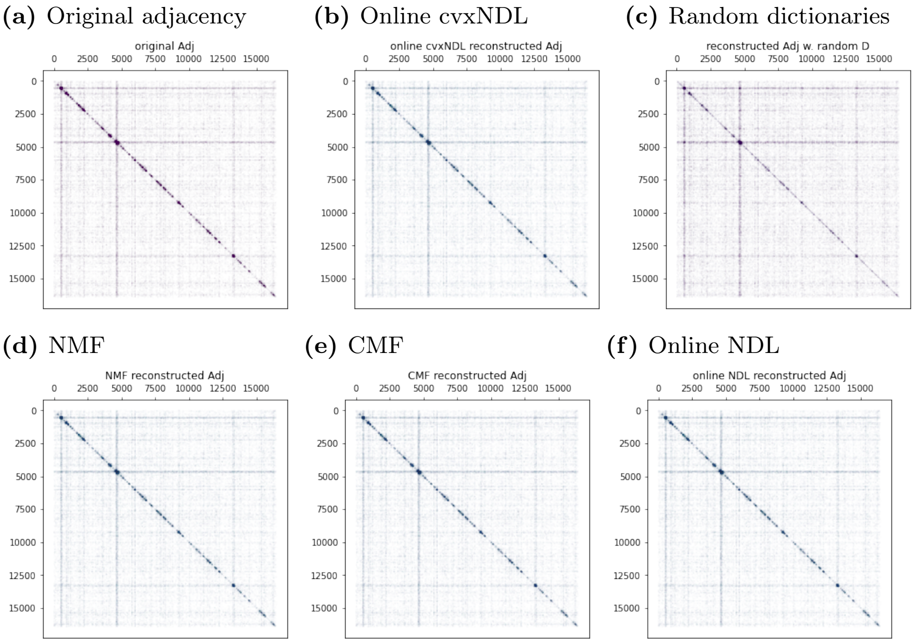

Fig J. Reconstructed network comparisons based on different baseline methods and random dictionaries, applied on *Drosophila* chromosome 2R. (a): The original adjacency matrix. (b, c, d, e, f): Reconstructed network adjacency matrices with online cxvNDL, random dictionary elements, NMF, CMF and online NDL.

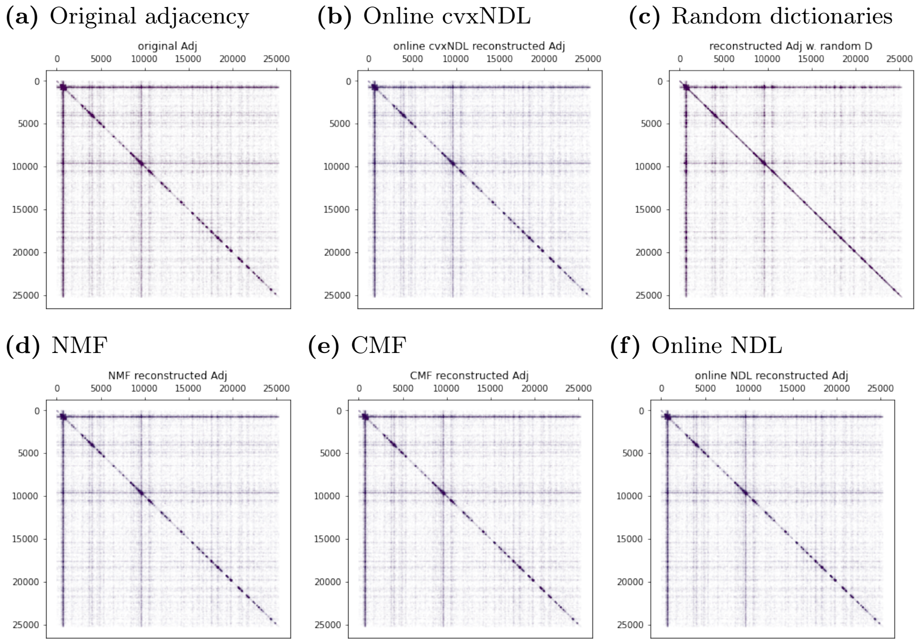

Fig K. Reconstructed network comparisons based on different baseline methods and random dictionaries, applied on *Drosophila* chromosome 3L. (a): The original adjacency matrix. (b, c, d, e, f): Reconstructed network adjacency matrices with online cxvNDL, random dictionary elements, NMF, CMF and online NDL.

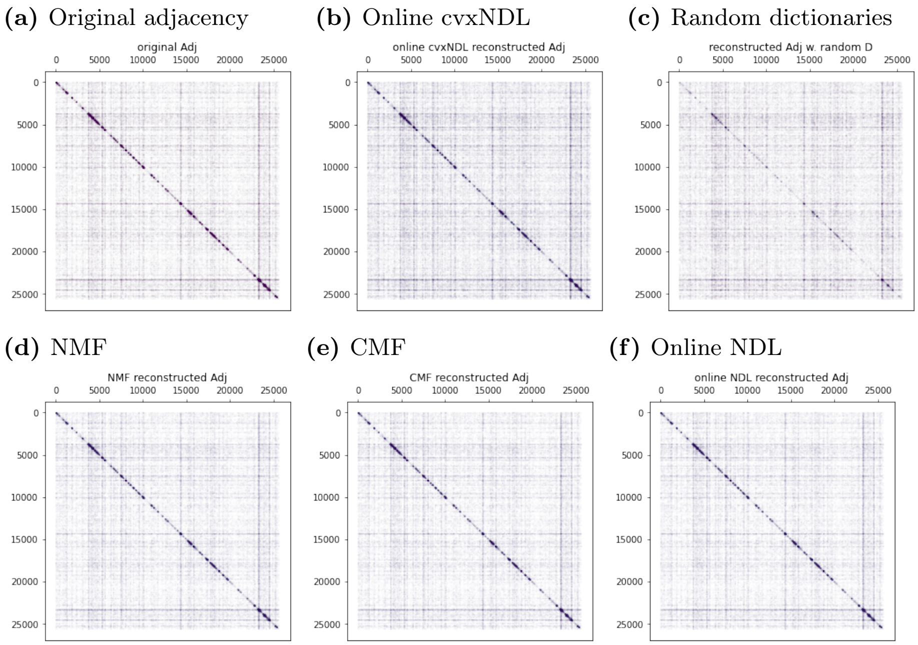

Fig L. Reconstructed network comparisons based on different baseline methods and random dictionaries, applied on *Drosophila* chromosome 3R. (a): The original adjacency matrix. (b, c, d, e, f): Reconstructed network adjacency with online cxvNDL, random dictionary elements, NMF, CMF and online NDL.

F Gene Ontology Enrichment Analysis

To associate a biological function with each dictionary element, we performed a gene ontology (GO) enrichment analysis for each element and the corresponding chromosome. Recall that as a results of the convexity constraint, every dictionary element has its corresponding set of representatives that capture real observed subgraphs which can be mapped back to actual genomic locations. Of most interest is the set of genes that covers at least one vertex in at least one of the representatives, as described in Fig M.

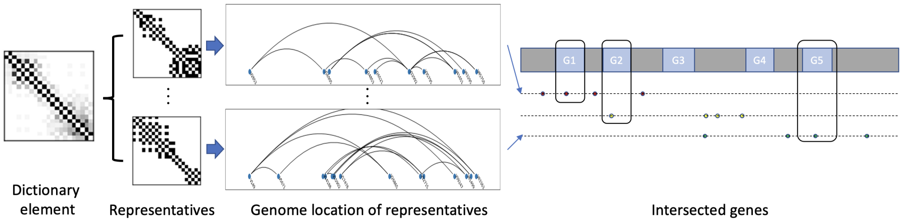

Fig M. GO enrichment analysis workflow. Each dictionary element is associated with a collection of real subnetwork representatives. These comprise nodes that can be mapped to the genome to identify their locations. A gene is said to cover the node if the genomic fragment corresponding to the node is fully contained within the gene.

Using the set of representative genes, we run the GO enrichment analysis using the annotation category "Biological Process" from `http://geneontology.org`, with the reference list *Drosophila Melanogaster* for each dictionary element. For further analysis, we only selected results with false discovery rate (FDR) < 0.05 and hence obtained candidate sets of enriched GO terms. Note that there may be inherently enriched GO terms for each dictionary element due to the sampling bias. To remove this bias, we ran another GO enrichment analysis with all genes on each chromosome and used those results to filter out the background GO terms for each dictionary element.

Furthermore, we utilized the hierarchical structure of GO terms [5], where terms are represented as nodes in a directed acyclic graph, and their relationships are described via arcs in the digraph. A child GO term is considered more specific than a parent GO term. Since the GO graph is not a strict hierarchy (a child node may have multiple parent nodes), to further improve the results, we performed the following processing. For each GO term: i) we first find all the paths between the term and the root node (which is "Biological process" in our setting), and ii) we remove all intermediate parent GO terms from its enriched GO terms set. By iteratively repeating this filtering process for each dictionary element, we derived a set of the most specific GO terms for each dictionary element.

F.1 Dictionary Elements Associated with GO Terms

We investigated the most frequently enriched GO terms as well as the least frequently enriched GO terms for each chromosome and identified the corresponding dictionary elements where they were found to be enriched. The results are shown in Tables D to G. For each dictionary element, we computed its density (complexity) ρ via $\rho = \frac{1}{k^2}\sum_{i,j}\mathbf{D}_{i,j}$ and the median genomic distance between all consecutive pairs of nodes, denoted by d_{med}. The full set of results for the densities and median distances for all dictionary elements and all chromosomes is provided in Tables B and C.

Note that the *Drosophila* S2 cells are embryonic cells, and most GO terms found are related to cellular reproductive process or developmental process, as expected. From the tables, one can also see that different dictionary elements reflect different biological processes and for the same GO term, the dictionary elements share similar patterns. For example, in Table D, we can see that dictionary elements 19 and 12 share very similar structural patterns, and both of them are enriched in biosynthetic processes of antibacterial peptides. On the other hand, dictionary elements 13 and 8 have a pattern that differs from that of 19 and 12, and they are enriched in dorsal/ventral lineage restriction processes. We also found that dictionary elements with GO term *peripheral nervous system development*, *celluar response to organic substance*, and *neuroblast fate determination* have relatively lower density and smaller median node distances than the top 2 enriched GO terms, *regulation of reproductive process* and *muscle cell cellular homeostasis*. The difference in density and median distance is also reflected by the significantly different dictionary patterns observed, such as dictionary element 12 and dictionary element 5; the former element has a much higher density and median distance than the latter.

There are also a few shared GO terms that are enriched in both chr2L and chr2R (11 shared terms in total) and in both chr3L and chr3R (3 shared terms in total). The results are reported in Table H and I. We found that there are very few shared terms between the two chromosomes when compared to the roughly one hundred uniquely enriched GO terms for each chromosome. Most of the shared terms also have "similar" patterns (which can be seen visually or through a simple computation of the ℓ_2 distance between their flattened adjacency matrices) of their corresponding dictionary elements.

Table D. The 5 most and least enriched GO terms within the span of dictionary elements for chr2L. Column '#' indicates the number of dictionary elements that show enrichment for the given GO term. Also reported are up to 3 dictionary elements with the largest importance score in the dictionary, along with the "density" ρ of interactions in the dictionary element and median distance d_{med} of all adjacent pairs of nodes in its representatives.

most frequent GO term	#	top 3 dictionaries	least frequent GO term	#	dictionary
(GO:2000241) regulation of reproductive process	5	dict_2 (0.085) dict_21 (0.070) dict_6 (0.044) 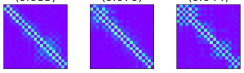 ρ=0.134,0.142,0.161 d_{med}=9906,8105,10024	(GO:0007485) imaginal disc-derived male genitalia development	1	dict_21 (0.070) 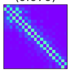 ρ=0.142 d_{med}= 8105
(GO:0046716) muscle cell cellular homeostasis	4	dict_14 (0.055) dict_6 (0.044) dict_12 (0.029) 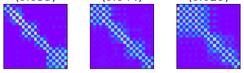 ρ=0.141,0.161,0.203 d_{med}=10928,10024,9979	(GO:0008347) glial cell migration	1	dict_5 (0.074) 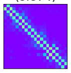 ρ=0.132 d_{med}= 8547
(GO:0007422) peripheral nervous system development	3	dict_5 (0.074) dict_7 (0.061) dict_8 (0.057) 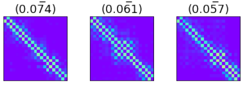 ρ=0.132,0.158,0.147 d_{med}=8547,8870,10692	(GO:0002920) regulation of humoral immune response	1	dict_21 (0.070) 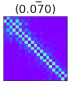 ρ=0.142 d_{med}= 8105
(GO:0071310) cellular response to organic substance	3	dict_2 (0.085) dict_21 (0.070) dict_7 (0.061) 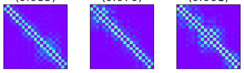 ρ=0.134,0.142,0.158 d_{med}=9906,8105,8870	(GO:0016075) rRNA catabolic process	1	dict_8 (0.057) 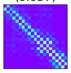 ρ=0.147 d_{med}= 10692
(GO:0007400) neuroblast fate determination	3	dict_5 (0.074) dict_21 (0.070) dict_8 (0.057) 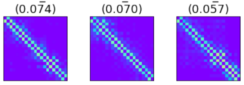 ρ=0.132,0.142,0.147 d_{med}=8547,8105,10692	(GO:0008258) head involution	1	dict_8 (0.057) 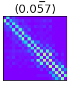 ρ=0.147 d_{med}= 10692

Table E. The 5 most and least enriched GO terms within the span of dictionary elements for chr2R. Column '#' indicates the number of dictionary elements that show enrichment for the given GO term. Also reported are up to 3 dictionary elements with the largest importance score in the dictionary, along with the "density" ρ of interactions in the dictionary element and median distance d_{med} of all adjacent pairs of nodes in its representatives.

most frequent GO term	#	top 3 dictionaries	least frequent GO term	#	dictionary
(GO:0030706) germarium-derived oocyte differentiation	6	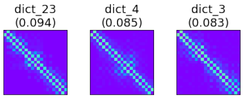<br>ρ=0.140,0.145,0.146<br>d_{med}=8764,7651,7158	(GO:0050803) regulation of synapse structure or activity	1	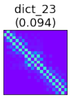<br>ρ=0.140<br>d_{med}= 8764
(GO:0001700) embryonic development via the syncytial blastoderm	5	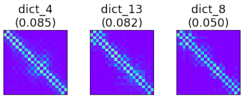<br>ρ=0.145,0.141,0.136<br>d_{med}=7651,8251,7085	(GO:0007498) mesoderm development	1	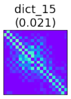<br>ρ=0.183<br>d_{med}= 7143
(GO:0007451) dorsal/ventral lineage restriction, imaginal disc	4	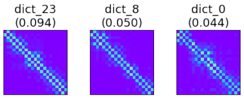<br>ρ=0.140,0.136,0.157<br>d_{med}=8764,7085,6738	(GO:0010638) positive regulation of organelle organization	1	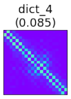<br>ρ=0.145<br>d_{med}= 7651
(GO:0006964) positive regulation of biosynthetic process of antibacterial peptides active against Gram-negative bacteria	3	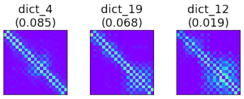<br>ρ=0.145,0.156,0.202<br>d_{med}=7651,8199,7706	(GO:0043277) apoptotic cell clearance	1	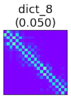<br>ρ=0.136<br>d_{med}= 7085
(GO:0045476) nurse cell apoptotic process	3	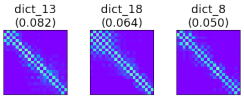<br>ρ=0.141,0.159,0.136<br>d_{med}=8251,7882,7085	(GO:0001707) mesoderm formation	1	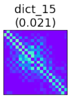<br>ρ=0.183<br>d_{med}= 7143

Table F. The 5 most and least enriched GO terms within the span of dictionary elements for chr3L. Column '#' indicates the number of dictionary elements that show enrichment for the given GO term. Also reported are up to 3 dictionary elements with the largest importance score in the dictionary, along with the "density" ρ of interactions in the dictionary element and median distance d_{med} of all adjacent pairs of nodes in its representatives.

most frequent GO term	#	top 3 dictionaries	least frequent GO term	#	dictionary
(GO:0009631) cold acclimation	2	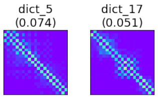 ρ=0.148,0.152 d_{med}=10608,8558	(GO:0035070) salivary gland histolysis	1	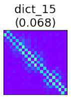 ρ=0.143 d_{med}= 8849
(GO:0009408) response to heat	2	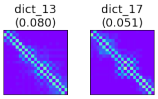 ρ=0.147,0.152 d_{med}=8689,8558	(GO:0046843) dorsal appendage formation	1	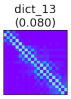 ρ=0.147 d_{med}= 8689
(GO:0007616) long-term memory	2	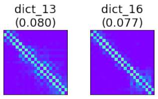 ρ=0.147,0.126 d_{med}=8689,9978	(GO:0007097) nuclear migration	1	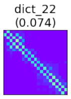 ρ=0.134 d_{med}= 11012
(GO:0061077) chaperone-mediated protein folding	2	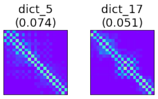 ρ=0.148,0.152 d_{med}=10608,8558	(GO:0035071) salivary gland cell autophagic cell death	1	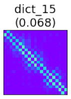 ρ=0.143 d_{med}= 8849
(GO:0008587) imaginal disc-derived wing margin morphogenesis	2	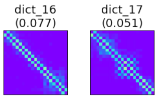 ρ=0.126,0.152 d_{med}=9978,8558	(GO:0007528) neuromuscular junction development	1	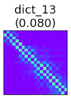 ρ=0.147 d_{med}= 8689

Table G. The 5 most and least enriched GO terms within the span of dictionary elements for chr3R. Column '#' indicates the number of dictionary elements that show enrichment for the given GO term. Also reported are up to 3 dictionary elements with the largest importance score in the dictionary, along with the "density" ρ of interactions in the dictionary element and median distance d_{med} of all adjacent pairs of nodes in its representatives.

most frequent GO term	#	top 3 dictionaries	least frequent GO term	#	dictionary
(GO:0001819) positive regulation of cytokine production	7	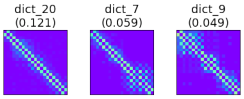 ρ=0.126,0.146,0.157 d_{med}=12791,12830,11930	(GO:0061448) connective tissue development	1	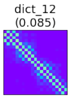 ρ=0.142 d_{med}= 13455
(GO:0008015) blood circulation	7	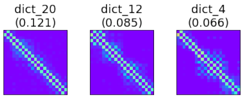 ρ=0.126,0.142,0.138 d_{med}=12791,13455,13674	(GO:0051282) regulation of sequestering of calcium ion	1	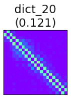 ρ=0.126 d_{med}= 12791
(GO:0045948) positive regulation of translational initiation	5	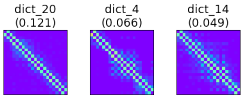 ρ=0.126,0.138,0.162 d_{med}=12791,13674,12572	(GO:0043123) positive regulation of I-kappaB kinase/NF-kappaB signaling	1	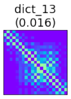 ρ=0.204 d_{med}= 12540
(GO:0042177) negative regulation of protein catabolic process	5	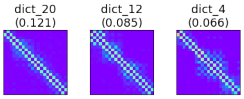 ρ=0.126,0.142,0.138 d_{med}=12791,13455,13674	(GO:0007435) salivary gland morphogenesis	1	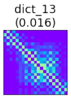 ρ=0.204 d_{med}= 12540
(GO:0043065) positive regulation of apoptotic process	4	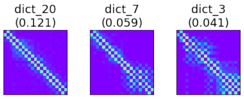 ρ=0.126,0.146,0.179 d_{med}=12791,12830,11748	(GO:0045738) negative regulation of DNA repair	1	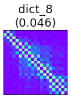 ρ=0.183 d_{med}= 12493

Table H. GO terms shared between chr2L and chr2R.

GO_term	chr2L dictionaries	chr2R dictionaries
(GO:0016325) oocyte microtubule cytoskeleton organization	dict_5 (0.074) dict_7 (0.061) dict_6 (0.044) 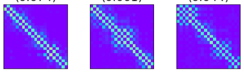	dict_14 (0.013) 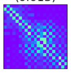
(GO:1901701) cellular response to oxygen-containing compound	dict_2 (0.085) dict_7 (0.061) 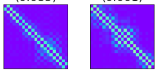	dict_8 (0.050) 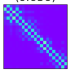
(GO:0007298) border follicle cell migration	dict_2 (0.085) dict_21 (0.070) 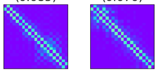	dict_4 (0.085) dict_3 (0.083) dict_18 (0.064) 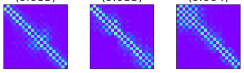
(GO:0043410) positive regulation of MAPK cascade	dict_2 (0.085) dict_8 (0.057) 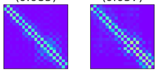	dict_4 (0.085) dict_8 (0.050) 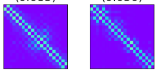
(GO:0016049) cell growth	dict_21 (0.070) 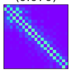	dict_8 (0.050) 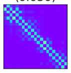
(GO:0035331) negative regulation of hippo signaling	dict_8 (0.057) 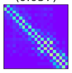	dict_4 (0.085) 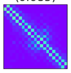
(GO:0051962) positive regulation of nervous system development	dict_7 (0.061) 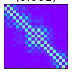	dict_15 (0.021) 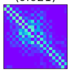
(GO:0060322) head development	dict_8 (0.057) 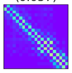	dict_4 (0.085) 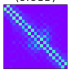
(GO:0007293) germarium-derived egg chamber formation	dict_8 (0.057) 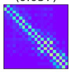	dict_23 (0.094) dict_4 (0.085) dict_13 (0.082) dict_15 (0.021) 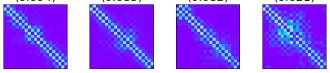
(GO:0002164) larval development	dict_6 (0.044) 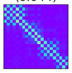	dict_15 (0.021) 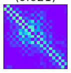
(GO:0007420) brain development	dict_6 (0.044) 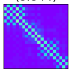	dict_4 (0.085) dict_18 (0.064) 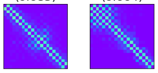

Table I. GO terms shared between chr3L and chr3R.

GO_term	chr3L dictionaries	chr3R dictionaries
(GO:0070373) negative regulation of ERK1 and ERK2 cascade	dict_13 (0.080) 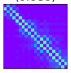 dict_22 (0.074) 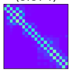 dict_3 (0.045) 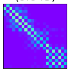 dict_1 (0.035) 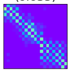	dict_8 (0.046) 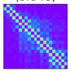
(GO:0007140) male meiotic nuclear division	dict_23 (0.029) 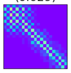	dict_24 (0.017) 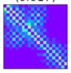
(GO:0046777) protein autophosphorylation	dict_22 (0.074) 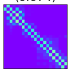	dict_8 (0.046) 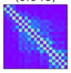

F.2 Additional Results

Here we report more detailed results for each dictionary element, including its number of enriched GO terms and importance scores (Tables J, K, L, M).

Table J. Number of enriched GO terms for each dictionary element identified for chr2L.

# GO terms	# GO terms	# GO terms	# GO terms	# GO terms
dict_0 (0.077) 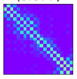 2	dict_5 (0.074) 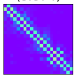 15	dict_10 (0.018) 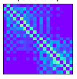 0	dict_15 (0.038) 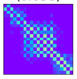 0	dict_20 (0.024) 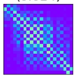 0
dict_1 (0.019) 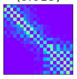 0	dict_6 (0.044) 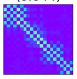 19	dict_11 (0.022) 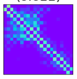 2	dict_16 (0.030) 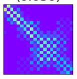 2	dict_21 (0.070) 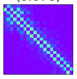 27
dict_2 (0.085) 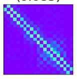 20	dict_7 (0.061) 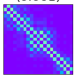 24	dict_12 (0.029) 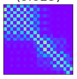 1	dict_17 (0.045) 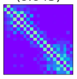 0	dict_22 (0.046) 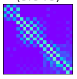 1
dict_3 (0.030) 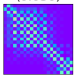 0	dict_8 (0.057) 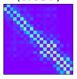 31	dict_13 (0.014) 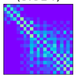 0	dict_18 (0.030) 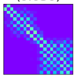 0	dict_23 (0.014) 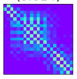 0
dict_4 (0.059) 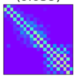 0	dict_9 (0.017) 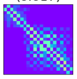 0	dict_14 (0.055) 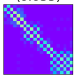 6	dict_19 (0.016) 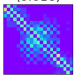 0	dict_24 (0.025) 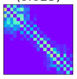 0

Table K. Number of enriched GO terms for each dictionary element identified for chr2R.

	# GO terms		# GO terms		# GO terms		# GO terms		# GO terms
dict_0 (0.044)	4	dict_5 (0.014)	0	dict_10 (0.014)	0	dict_15 (0.021)	23	dict_20 (0.041)	6
dict_1 (0.041)	1	dict_6 (0.025)	0	dict_11 (0.042)	1	dict_16 (0.018)	0	dict_21 (0.019)	0
dict_2 (0.035)	0	dict_7 (0.037)	1	dict_12 (0.019)	2	dict_17 (0.020)	0	dict_22 (0.019)	8
dict_3 (0.083)	12	dict_8 (0.050)	17	dict_13 (0.082)	9	dict_18 (0.064)	8	dict_23 (0.094)	10
dict_4 (0.085)	40	dict_9 (0.030)	0	dict_14 (0.013)	5	dict_19 (0.068)	7	dict_24 (0.022)	2

Table L. Number of enriched GO terms for each dictionary element identified for chr3L.

	# GO terms		# GO terms		# GO terms		# GO terms		# GO terms
dict_0 (0.022)	0	dict_5 (0.074)	6	dict_10 (0.023)	2	dict_15 (0.068)	10	dict_20 (0.025)	0
dict_1 (0.035)	3	dict_6 (0.028)	1	dict_11 (0.027)	0	dict_16 (0.077)	14	dict_21 (0.018)	0
dict_2 (0.049)	0	dict_7 (0.029)	1	dict_12 (0.021)	1	dict_17 (0.051)	9	dict_22 (0.074)	4
dict_3 (0.045)	3	dict_8 (0.020)	0	dict_13 (0.080)	16	dict_18 (0.023)	4	dict_23 (0.029)	3
dict_4 (0.074)	3	dict_9 (0.023)	0	dict_14 (0.009)	0	dict_19 (0.037)	0	dict_24 (0.040)	0

Table M. Number of enriched GO terms for each dictionary element identified for chr3R.

	# GO terms		# GO terms		# GO terms		# GO terms		# GO terms
dict_0 (0.046) 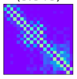	15	dict_5 (0.038) 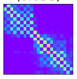	2	dict_10 (0.040) 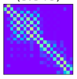	5	dict_15 (0.016) 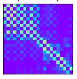	8	dict_20 (0.121) 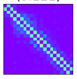	124
dict_1 (0.042) 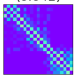	9	dict_6 (0.029) 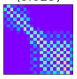	2	dict_11 (0.021) 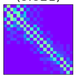	0	dict_16 (0.019) 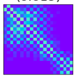	0	dict_21 (0.041) 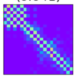	10
dict_2 (0.062) 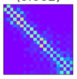	13	dict_7 (0.059) 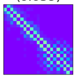	14	dict_12 (0.085) 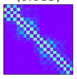	16	dict_17 (0.015) 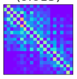	0	dict_22 (0.022) 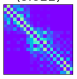	4
dict_3 (0.041) 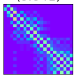	7	dict_8 (0.046) 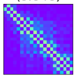	25	dict_13 (0.016) 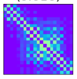	57	dict_18 (0.014) 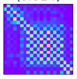	0	dict_23 (0.016) 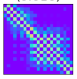	0
dict_4 (0.066) 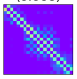	20	dict_9 (0.049) 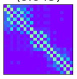	1	dict_14 (0.049) 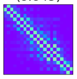	6	dict_19 (0.027) 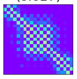	0	dict_24 (0.017) 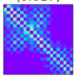	4

G RNA-Seq Coexpression Analysis

The ChIA-Drop dataset [9] used for learning dictionaries of chromatin interactions lacks RNA-Seq replicates, posing a challenge when trying to validate our results through coexpression analysis. To address this limitation, we retrieved RNA-Seq data corresponding to untreated S2 cell lines of *Drosophila Melanogaster* from the Digital Expression Explorer (DEE2) repository. DEE2 provides uniformly processed RNA-Seq data sourced from the publicly available NCBI Sequence Read Archive (SRA) [10]. In total, we retrieved 20 samples from untreated S2 cell lines with their IDs reported in Table N.

Table N. Sample IDs retrieved from NCBI Sequence Read Archive for RNA-Seq coexpression analysis.

SRR12191916	SRR12191917	SRR12191918	SRR12191920	SRR12191921
SRR12191923	SRR12191927	SRR2442878	SRR2442879	SRR3065067
SRR5340065	SRR5340066	SRR5340069	SRR5340070	SRR5340071
SRR5340072	SRR6930637	SRR8108628	SRR8108629	SRR8108630

To ensure consistent normalization across all samples, we use the trimmed mean of M values (TMM) method [7], available through the edgeR package [6]. This is of crucial importance when jointly analyzing samples from multiple sources. We selected the most relevant genes by filtering the list of covered genes and retaining only those with more than 95% overlap with the gene promoter regions, as defined in the *Ensmbl* browser. Subsequently, for each dictionary element, we collected all genes covered by it and calculated the pairwise Pearson correlation coefficient of expressions of pairs of genes in the set. For a pair of random variables X_1 and X_2, the correlation coefficient is defined as

$$\rho_{X_1X_2} = \frac{\text{Covariance}(X_1, X_2)}{\text{Var}(X_1)\text{Var}(X_2)}$$

For two genes G_1 and G_2, let X_1 and X_2 be vectors of normalized read counts. The Pearson correlation coefficient can be written as

$$\rho_{G_1G_2} = \frac{\sum_{i=1}^{n}(x_{1i} - \bar{x}_1)(x_{2i} - \bar{x}_2)}{\sqrt{\sum_{i=1}^{n}(x_{1i} - \bar{x}_1)^2}\sqrt{\sum_{i=1}^{n}(x_{2i} - \bar{x}_2)^2}}$$

where

n is the number of samples,

$\bar{x}_1 = \frac{\sum_{i=1}^{n} x_{1i}}{n}$ and $\bar{x}_2 = \frac{\sum_{i=1}^{n} x_{2i}}{n}$ are sample means.

To visualize the underlying coexpression clusters within the genes, we performed hierarchical clustering. We report the mean correlation statistics as well as mean statistics for positively correlated genes for each dictionary element. Correlation plots for all dictionary elements are shown in Figs N, O, P and Q.

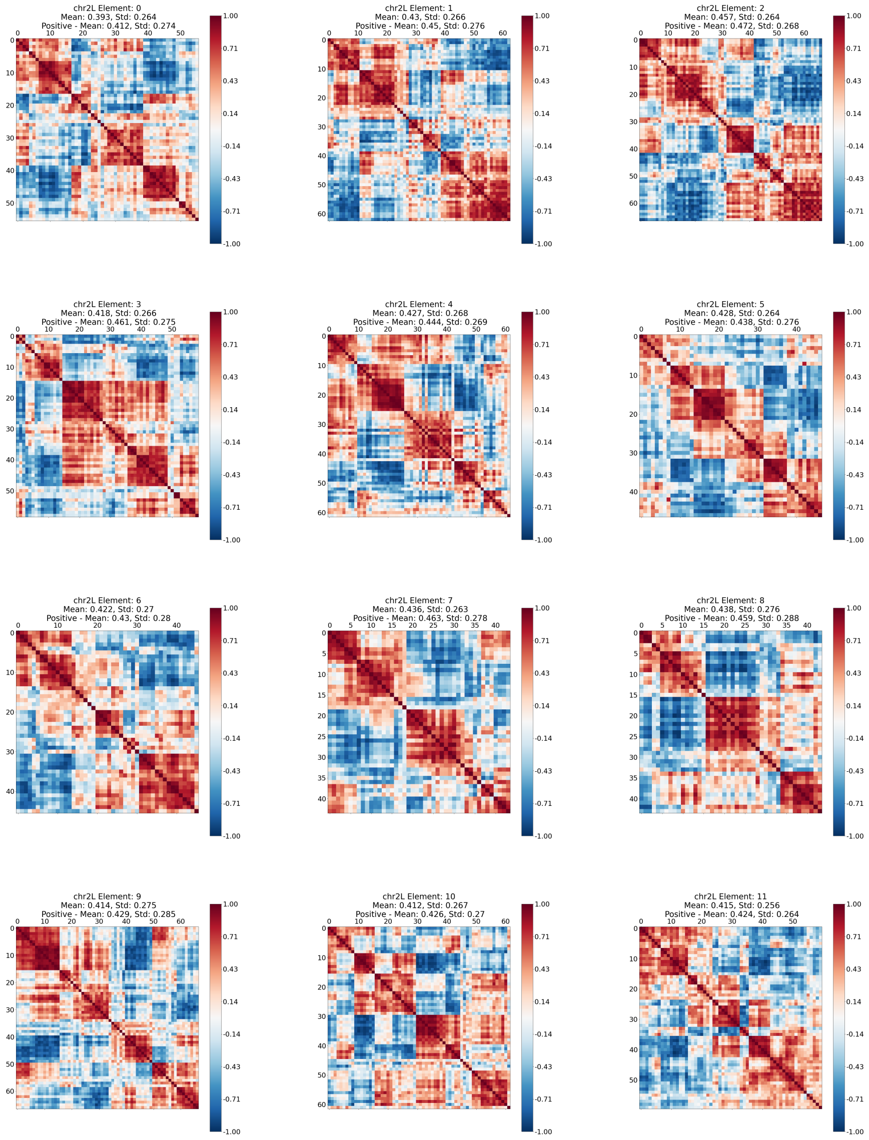

Fig N. Pairwise coexpression of genes covered by various dictionary elements for chr 2L obtained through online cvxNDL. We calculated the mean and standard deviation of absolute pairwise coexpression values, along with the mean and standard deviation of coexpression values specifically for all positively correlated gene pairs.

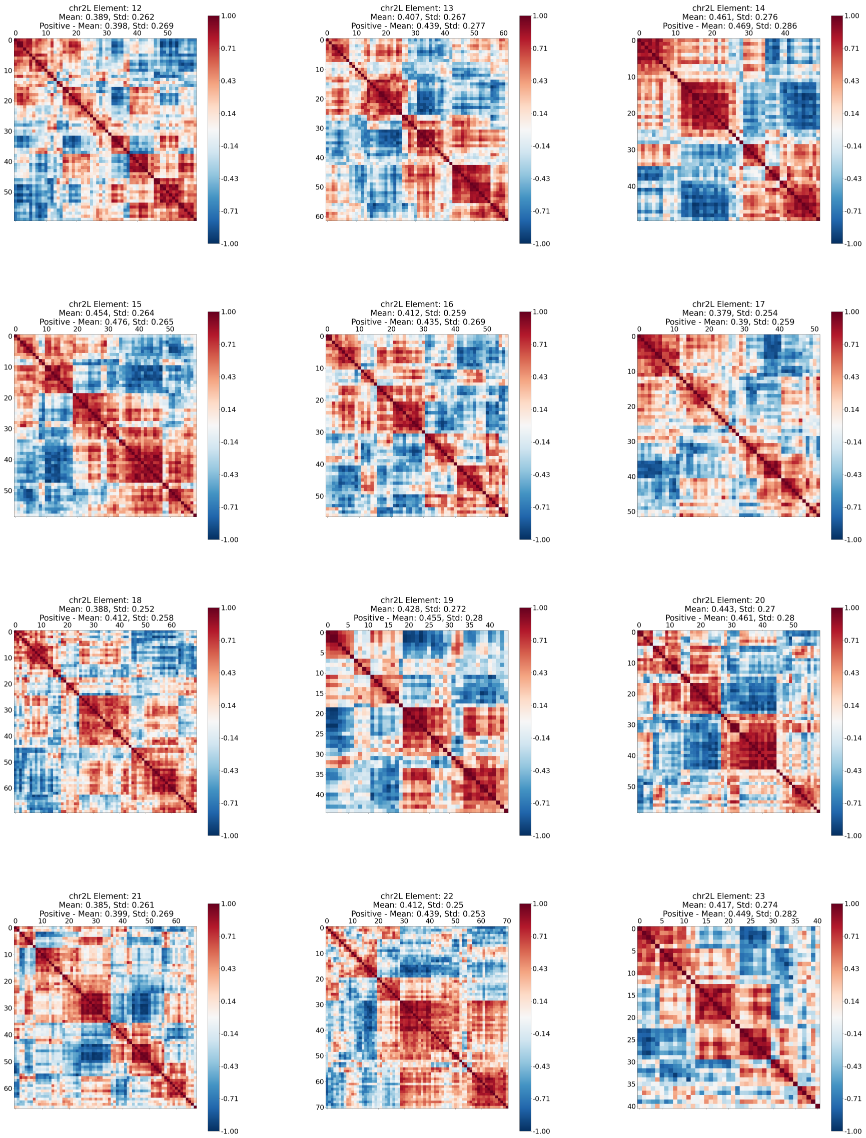

Fig N. Pairwise coexpression of genes covered by various dictionary elements for chr 2L obtained through online cvxNDL. We calculated the mean and standard deviation of absolute pairwise coexpression values, along with the mean and standard deviation of coexpression values specifically for all positively correlated gene pairs.

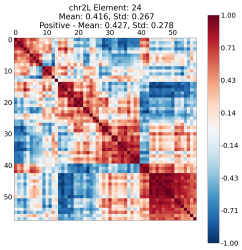

Fig N. Pairwise coexpression of genes covered by various dictionary elements for chr 2L obtained through online cvxNDL. We calculated the mean and standard deviation of absolute pairwise coexpression values, along with the mean and standard deviation of coexpression values specifically for all positively correlated gene pairs.

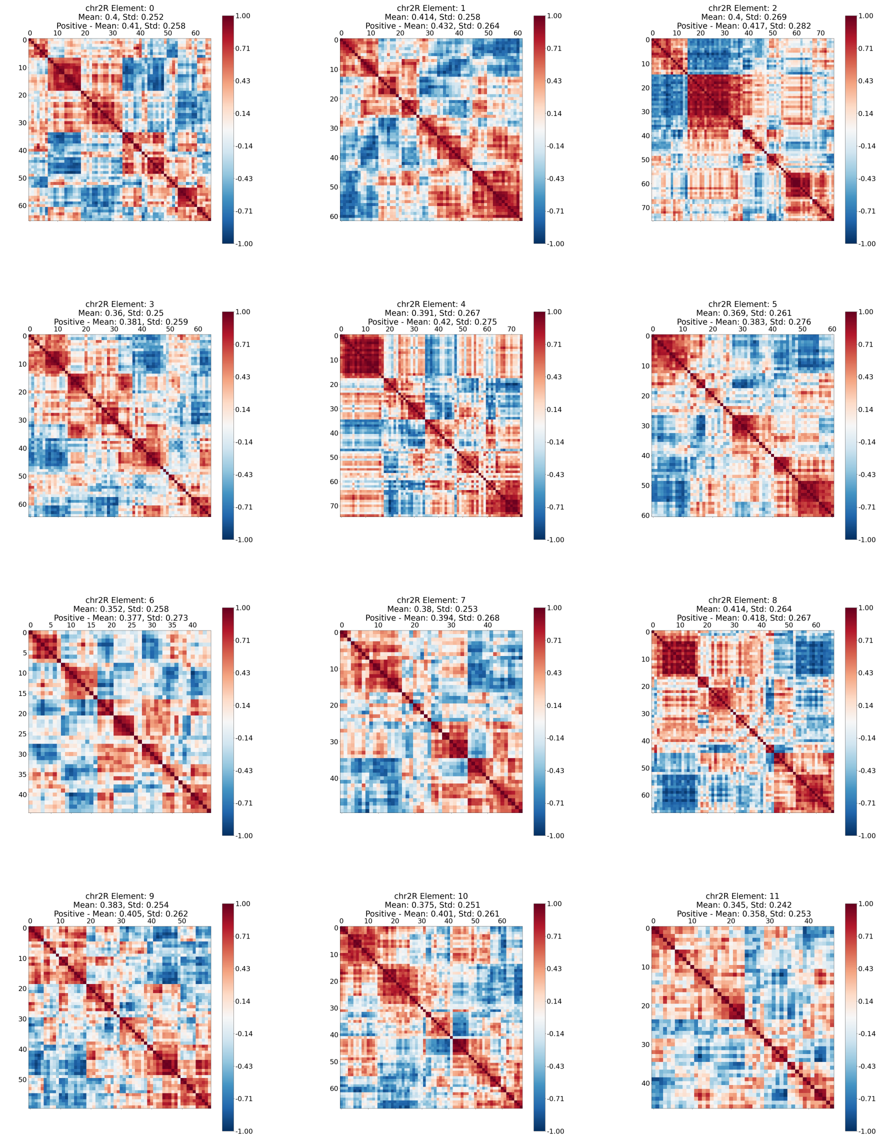

Fig O. Pairwise coexpression of genes covered by various dictionary elements for chr 2R obtained through online cvxNDL. We calculated the mean and standard deviation of absolute pairwise coexpression values, along with the mean and standard deviation of coexpression values specifically for all positively correlated gene pairs.

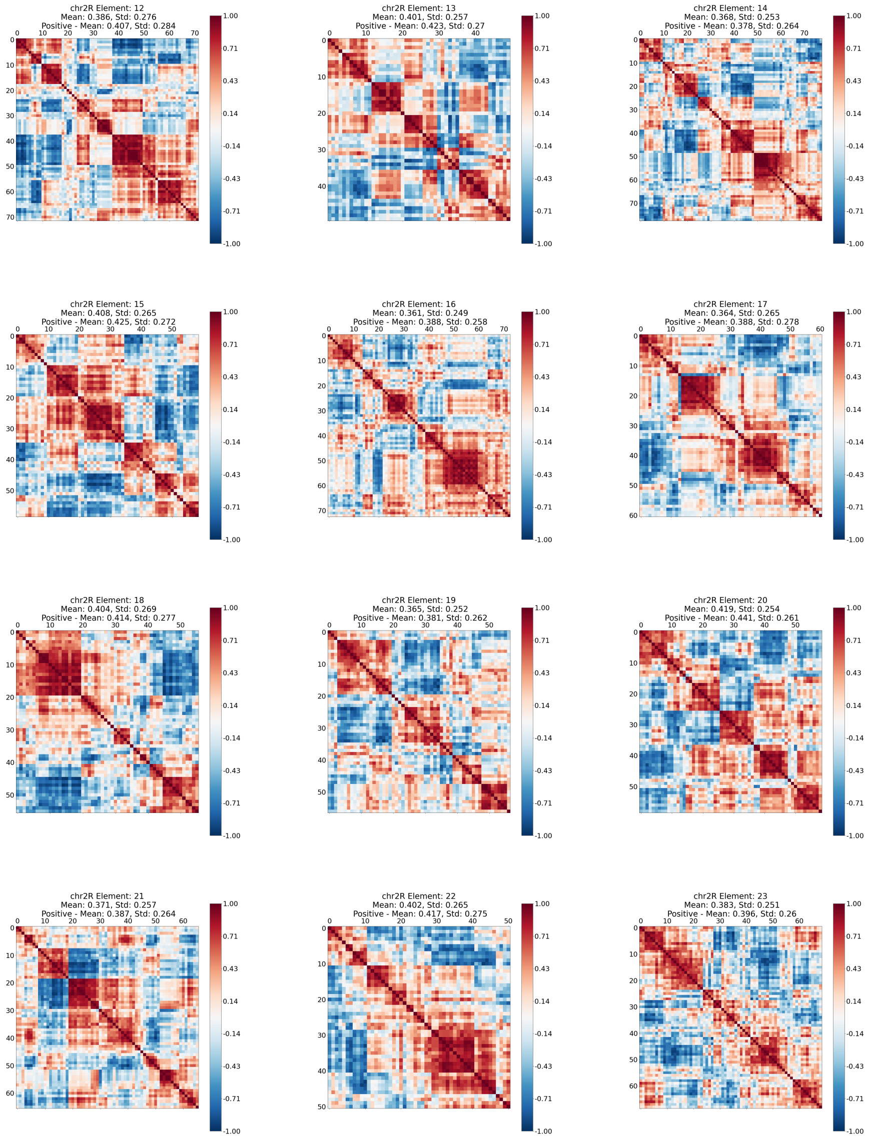

Fig O. Pairwise coexpression of genes covered by various dictionary elements for chr 2R obtained through online cvxNDL. We calculated the mean and standard deviation of absolute pairwise coexpression values, along with the mean and standard deviation of coexpression values specifically for all positively correlated gene pairs.

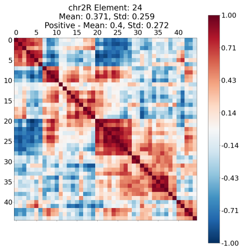

Fig O. Pairwise coexpression of genes covered by various dictionary elements for chr 2R obtained through online cvxNDL. We calculated the mean and standard deviation of absolute pairwise coexpression values, along with the mean and standard deviation of coexpression values specifically for all positively correlated gene pairs.

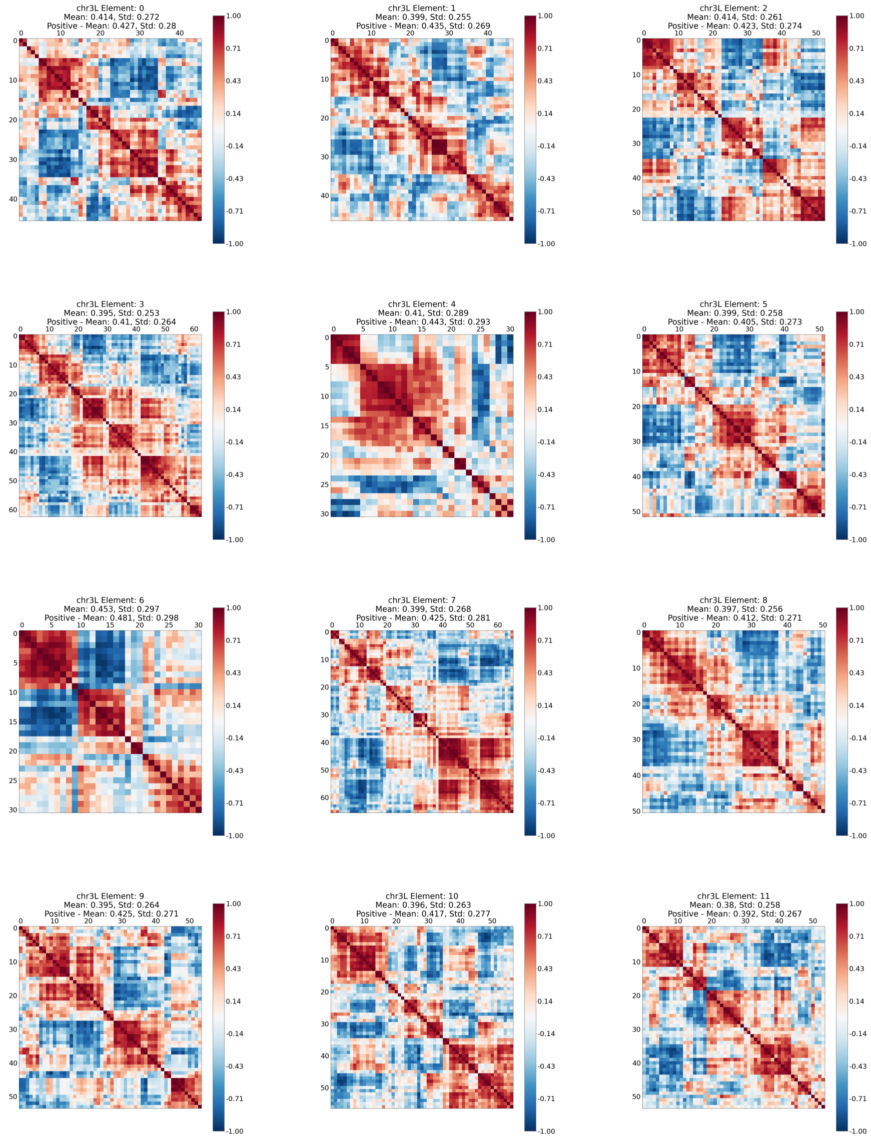

Fig P. Pairwise coexpression of genes covered by various dictionary elements for chr 3L obtained through online cvxNDL. We calculated the mean and standard deviation of absolute pairwise coexpression values, along with the mean and standard deviation of coexpression values specifically for all positively correlated gene pairs.

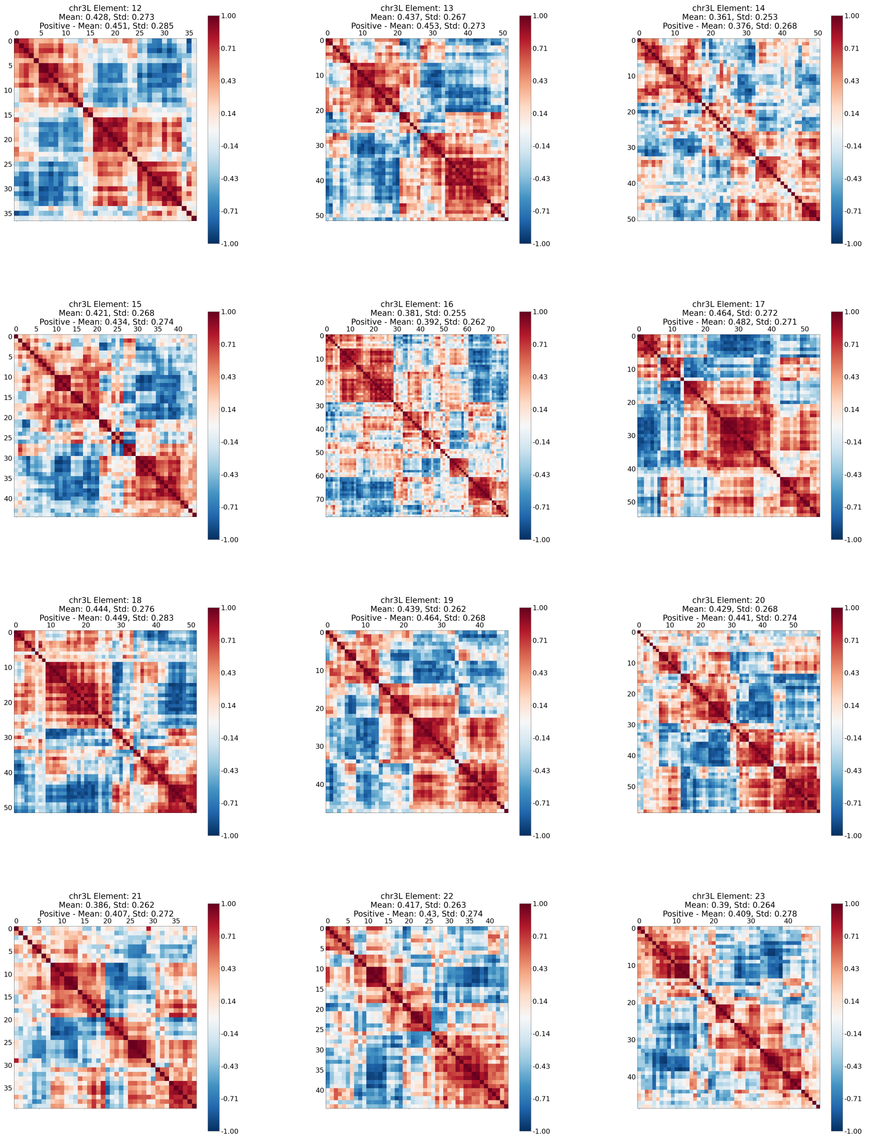

Fig P. Pairwise coexpression of genes covered by various dictionary elements for chr 3L obtained through online cvxNDL. We calculated the mean and standard deviation of absolute pairwise coexpression values, along with the mean and standard deviation of coexpression values specifically for all positively correlated gene pairs.

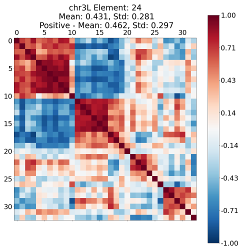

Fig P. Pairwise coexpression of genes covered by various dictionary elements for chr 3L obtained through online cvxNDL. We calculated the mean and standard deviation of absolute pairwise coexpression values, along with the mean and standard deviation of coexpression values specifically for all positively correlated gene pairs.

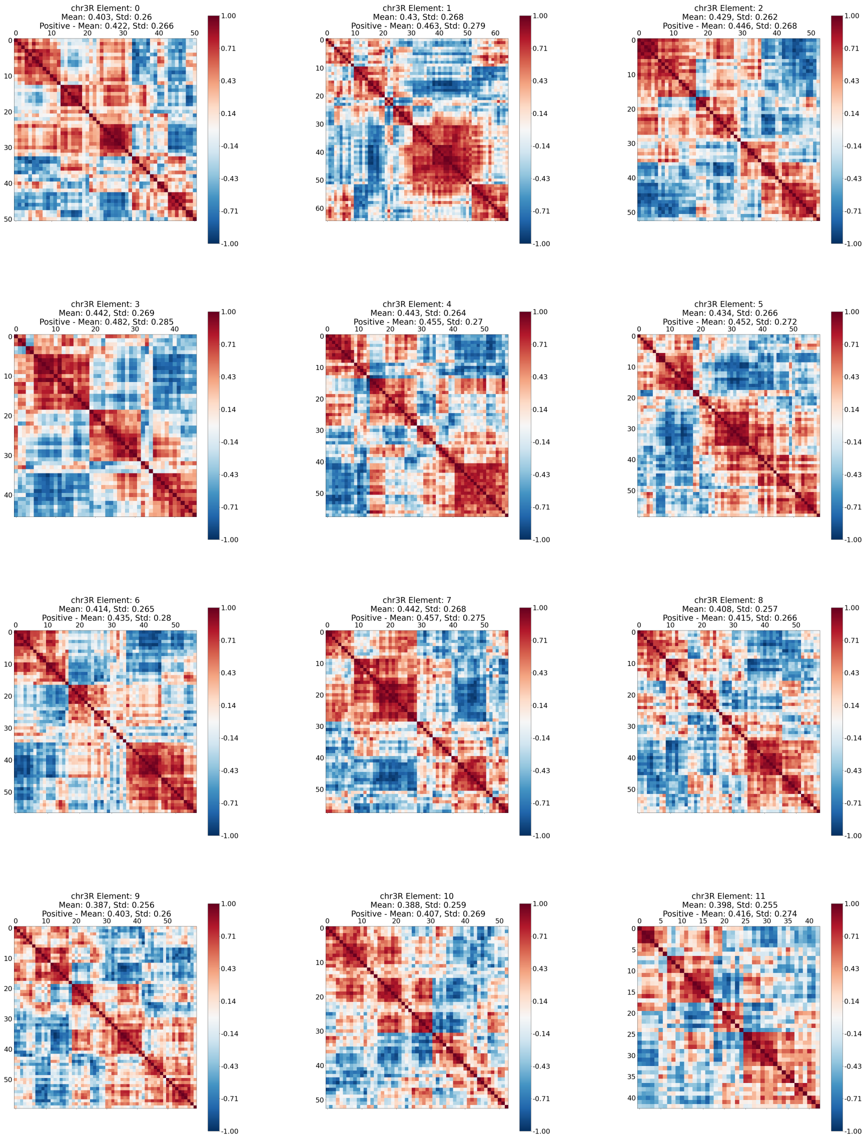

Fig Q. Pairwise coexpression of genes covered by various dictionary elements for chr 3R obtained through online cvxNDL. We calculated the mean and standard deviation of absolute pairwise coexpression values, along with the mean and standard deviation of coexpression values specifically for all positively correlated gene pairs.

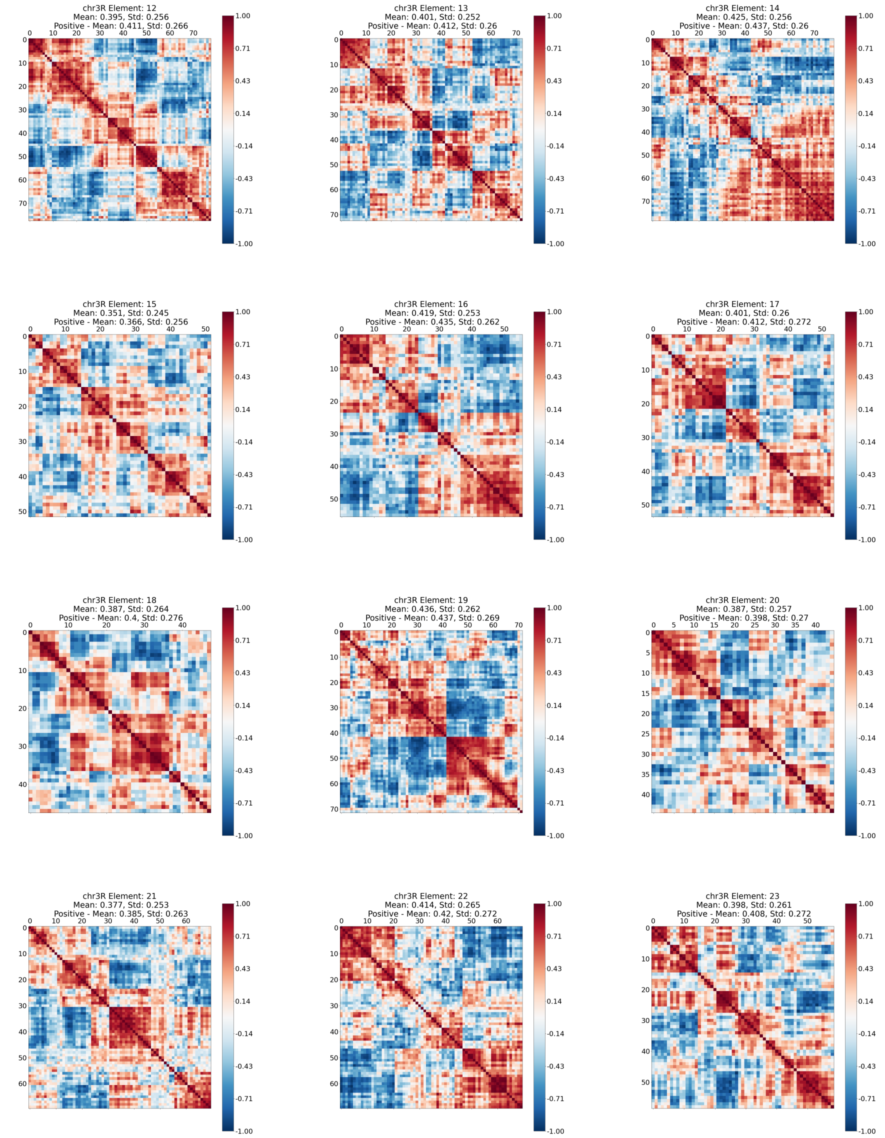

Fig Q. Pairwise coexpression of genes covered by various dictionary elements for chr 3R obtained through online cvxNDL. We calculated the mean and standard deviation of absolute pairwise coexpression values, along with the mean and standard deviation of coexpression values specifically for all positively correlated gene pairs.

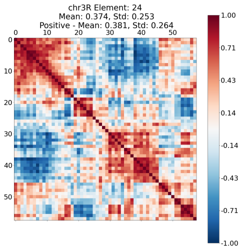

Fig Q. Pairwise coexpression of genes covered by various dictionary elements for chr 3R obtained through online cvxNDL. We calculated the mean and standard deviation of absolute pairwise coexpression values, along with the mean and standard deviation of coexpression values specifically for all positively correlated gene pairs.

H STRING interaction network and FlyMine

The STRING interaction network [8] provides a confidence score indicating the interaction likelihood between a pair of proteins within an organism. This score reflects both direct interactions via physical protein binding and indirect interactions by virtue of the proteins participating in the same cellular pathways. The confidence level of interaction between a pair of proteins can vary from 0, indicating very low confidence, to 1000, indicating very high confidence. Fig Ra shows the distribution of confidence levels between all pairs of proteins in the STRING database for *Drosophila Melanogaster*. A large majority of these interactions are very low confidence. To focus on more reliable interactions, we filtered the protein interactions to retain only those with a confidence score exceeding 200, resulting in a refined dataset shown in Fig Rb. By mapping these proteins back to their corresponding genes, we derived an induced network representing gene-gene interactions.

(a) All confidence values. **(b)** Confidence values filtered for > 200.

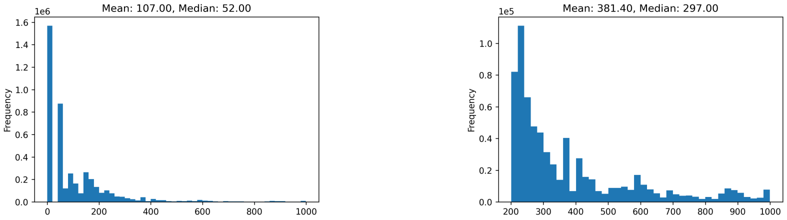

Fig R. Histogram of confidence values for pairwise interaction of proteins in the STRING interaction network for *Drosophila Melanogaster*.

For the online cvxNDL dictionary, we calculated the mean confidence level for all pairs of proteins. We also repeated the same experiments with a randomly constructed dictionary as a control. Fig S shows the mean confidence level and confidence interval for a subset of dictionary elements. We performed a K-S test with the null hypothesis that the two sets of confidence scores for pairwise interactions belonging to online cvxNDL dictionaries and randomly constructed dictionaries are drawn from the same distribution. We rejected the null hypothesis with p-value < 0.05. The mean confidence values of interactions (and the corresponding standard deviation) for all online cvxNDL and CMF dictionary elements and for each of the 4 chromosomes are shown in Table O.

Flymine [2] is a large genomic and proteomic database for *Drosophila*. We used FlyMine to retrieve a list of upregulated genes in S2 cell lines. We observe that the upregulated genes are overrepresented in our dictionary elements. To test our hypothesis, we performed the hypergeometric overrepresentation test. Our null hypothesis is that the proportion of upregulated genes in our dictionary elements is no higher than the overall proportion of upregulated genes in S2 cell lines. We rejected the null hypothesis (p-value < 0.05) for all dictionary elements for all chromosomes except a small subset of 4 dictionary elements (1 dictionary

(a) Mean confidence value for dictionary elements from chr2L and chr2R.

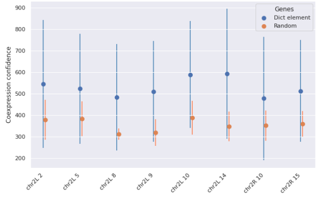

(b) Mean confidence value for dictionary elements from chr3L and chr3R

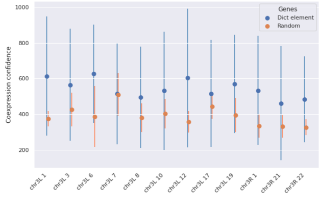

Fig S. Confidence levels for pairwise interaction of proteins for dictionary elements based on STRING interaction network.

Chromosome	Online cvxNDL	CMF
chr2L	0.424 (0.088)	0.405 (0.088)
chr2R	0.380 (0.060)	0.416 (0.084)
chr3L	0.426 (0.121)	0.445 (0.145)
chr3R	0.389 (0.076)	0.401 (0.074)

Table O. The mean confidence value of interactions based on STRING interaction network for all 25 online cvxNDL and CMF dictionary elements, and for each of the four chromosomes analyzed (and their standard deviation).

element from chr2R and 3 dictionary elements from chr3L). The p-values for all dictionary elements are shown in Table P.

Table P. Results for hypergeometric overrepresentation test for all dictionary elements. We report the p-values corresponding to the null hypothesis that the proportion of upregulated genes in our dictionary elements is no higher than the overall proportion of upregulated genes in S2 cell lines.

dictionary element	chr2L	chr2R	chr3L	chr3R
0	1.18E-03	5.90E-07	7.96E-05	3.24E-05
1	1.93E-08	8.13E-06	5.38E-04	9.23E-09
2	4.36E-08	4.44E-07	1.40E-03	1.36E-02
3	8.13E-06	7.92E-05	1.65E-04	4.49E-08
4	4.50E-06	1.83E-04	2.54E-03	4.88E-12
5	1.23E-06	3.93E-04	3.53E-03	5.84E-05
6	1.26E-03	2.88E-03	5.58E-03	6.07E-06
7	1.60E-03	3.88E-06	1.76E-03	1.39E-05
8	3.50E-05	9.15E-07	1.22E-04	3.03E-05
9	2.17E-04	2.17E-06	2.73E-04	4.36E-07
10	1.02E-05	3.57E-02	5.23E-06	2.37E-06
11	1.82E-05	8.94E-04	8.92E-02	1.96E-04
12	2.08E-06	8.90E-04	2.01E-01	3.23E-05
13	8.12E-05	8.52E-03	3.40E-05	1.73E-04
14	1.95E-05	1.41E-04	1.93E-03	1.84E-10
15	6.95E-08	5.78E-05	1.20E-02	8.32E-05
16	5.02E-03	7.60E-04	1.78E-03	4.82E-06
17	3.24E-04	5.41E-02	9.17E-06	7.53E-04
18	1.78E-03	6.04E-06	1.96E-02	3.89E-06
19	3.89E-04	3.56E-05	8.10E-04	6.86E-08
20	1.75E-08	2.90E-04	5.02E-03	1.50E-04
21	6.41E-03	1.55E-02	3.72E-06	8.88E-10
22	2.99E-03	1.40E-03	2.24E-05	9.23E-09
23	1.65E-05	6.78E-03	5.98E-03	3.42E-07
24	2.54E-06	1.03E-04	6.22E-02	7.19E-08

I Accessibility of figures

To help improve the accessibility of our work for people with colour blindness, we suggest using the Visolve software that can help interpret figures that may represent issues for readers who are colour blind. The Visolve software allows for easy interpretation of figures for people with various forms of color blindness and can be downloaded from [https://www.ryobi.co.jp/products/visolve/en/]. Here, we reproduce the dictionary elements corresponding to chr2L using CMF and online cvxNDL and their versions highlighting regions corresponding to a range of distances (colors) using Visolve.

(a) Online cvxNDL dictionaries with green filter

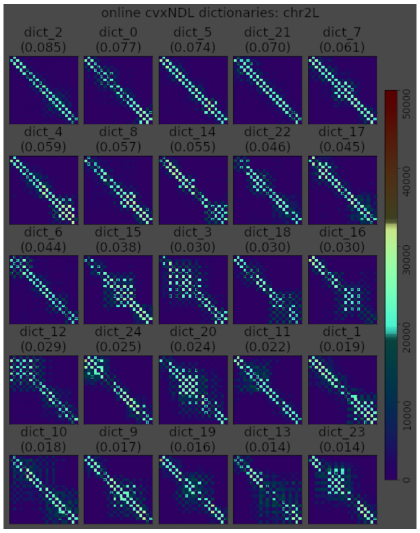

(b) CMF dictionaries with green filter

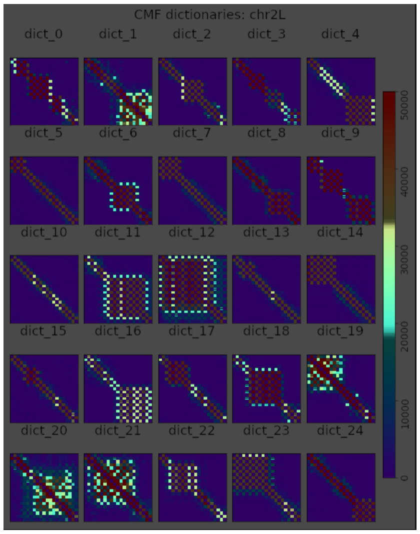

(c) Online cvxNDL dictionaries with red filter

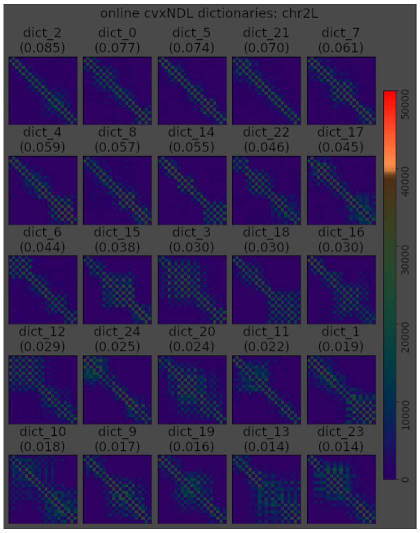

(d) CMF dictionaries with red filter

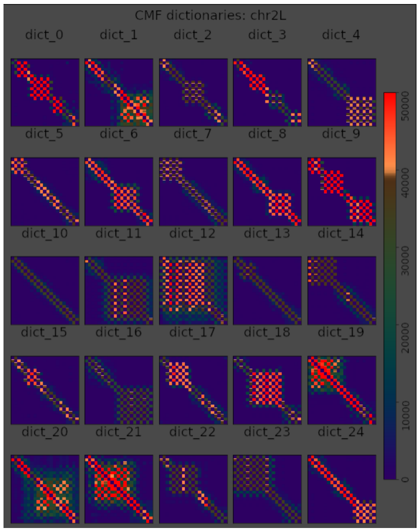

Fig T. Online cvxNDL and CMF dictionaries for chr2L with green ($20,000$ bases - $35,000$ bases) and red ($> 40,000$ bases) filters from Visolve software.

References

1. Paul W Holland, Kathryn Blackmond Laskey, and Samuel Leinhardt. Stochastic blockmodels: First steps. *Social networks*, 5(2):109–137, 1983.

2. Rachel Lyne, Richard Smith, Kim Rutherford, Matthew Wakeling, Andrew Varley, Francois Guillier, Hilde Janssens, Wenyan Ji, Peter Mclaren, Philip North, et al. Flymine: an integrated database for drosophila and anopheles genomics. *Genome biology*, 8(7):1–16, 2007.

3. Hanbaek Lyu, Facundo Memoli, and David Sivakoff. Sampling random graph homomorphisms and applications to network data analysis. *Journal of machine learning research*, 24(9):1–79, 2023.

4. Hanbaek Lyu, Deanna Needell, and Laura Balzano. Online matrix factorization for markovian data and applications to network dictionary learning. *Journal of Machine Learning Research*, 21(251):1–49, 2020.

5. Mark A Musen. The protégé project: a look back and a look forward. *AI matters*, 1(4):4–12, 2015.

6. Mark D Robinson, Davis J McCarthy, and Gordon K Smyth. edger: a bioconductor package for differential expression analysis of digital gene expression data. *bioinformatics*, 26(1):139–140, 2010.

7. Mark D Robinson and Alicia Oshlack. A scaling normalization method for differential expression analysis of rna-seq data. *Genome biology*, 11(3):1–9, 2010.

8. Damian Szklarczyk, Annika L Gable, David Lyon, Alexander Junge, Stefan Wyder, Jaime Huerta-Cepas, Milan Simonovic, Nadezhda T Doncheva, John H Morris, Peer Bork, et al. String v11: protein–protein association networks with increased coverage, supporting functional discovery in genome-wide experimental datasets. *Nucleic acids research*, 47(D1):D607–D613, 2019.

9. Meizhen Zheng, Simon Zhongyuan Tian, Daniel Capurso, Minji Kim, Rahul Maurya, Byoungkoo Lee, Emaly Piecuch, Liang Gong, Jacqueline Jufen Zhu, Zhihui Li, et al. Multiplex chromatin interactions with single-molecule precision. *Nature*, 566(7745):558–562, 2019.

10. Mark Ziemann, Antony Kaspi, and Assam El-Osta. Digital expression explorer 2: a repository of uniformly processed rna sequencing data. *Gigascience*, 8(4):giz022, 2019.
